# Clinical activity of a htert (vx-001) cancer vaccine as post-chemotherapy maintenance immunotherapy in patients with stage IV non-small cell lung cancer: final results of a randomised phase 2 clinical trial

**DOI:** 10.1038/s41416-020-0785-y

**Published:** 2020-03-25

**Authors:** Cesare Gridelli, Tudor Ciuleanu, Manuel Domine, Aleksandra Szczesna, Isabel Bover, Manuel Cobo, Nikolaos Kentepozidis, Konstantinos Zarogoulidis, Charalabos Kalofonos, Andrzej Kazarnowisz, Magdalena Korozan, Ramon de las Penas, Margarita Majem, Antonio Chella, Frank Griesinger, Evangelos Bournakis, Parvis Sadjadian, Athanasios Kotsakis, Thierry Chinet, Kostantinos N. Syrigos, Pierpaolo Correale, Catherine Gallou, Jeanne- Menez Jamet, Eleni- Kyriaki Vetsika, Kostas Kosmatopoulos, Vassilis Georgoulias, on behalf of the Vx-001-201 trial team

**Affiliations:** 1 S.G. Moscati Hospital, Avellino, Italy; 2 Institutui, Oncologic I. Chircuta, Cluz-Napoca, Romania; 3 Fundacion Jimenez Diaz, Madrid, Spain; 4 Mazowieckie Centrum, Otwock, Poland; 5 Son Llatzer Hospital, Palma de Mallorca, Spain; 6 Hospital Regional Universitario Málaga, Instituto de Investigaciones Biomédicas (IBIMA), Málaga, Spain; 7 251 General Airforce Hospital, Athens, Greece; 8 Papanikolaou General Hospital, Exohi, Greece; 9 University Hospital of Patras, Rio, Greece; 10 Oddzial Onkologiiz Pododdziatem, Olsztyn, Poland; 11 Hospicjum Dutkiewicza SAC, Gdansk, Poland; 12 Hospital Provincial de Castellon, Castellon, Spain; 13 Hospital de la Santa Creui Sant Pau, Barcelona, Spain; 14 A.O.U. di Pisa Hospital, Pisa, Italy; 15 Pius Hospital, Oldenburg, Germany; 16 University Hospital “Aretaieion”, Athens, Greece; 17 Johannes Wesling Klinikum, Minden, Germany; 18 Dpt of Medical Oncology, University General Hospital of Larissa, Larissa, Greece; 19 Hopital Ambroise Paré, Boulogne-Billancourt, France; 20 General Hospital of Thoracic Diseases ‘’Sotiria”, Athens, Greece; 21 University Hospital of Siena, Siena, Italy; 22 Vaxon-Biotech, Paris, France; 23 University General Hospital of Heraklion, Heraklion, Crete Greece

**Keywords:** Oncology, Non-small-cell lung cancer

## Abstract

**Background:**

The cancer vaccine Vx-001, which targets the universal tumour antigen TElomerase Reverse Transcriptase (TERT), can mount specific Vx-001/TERT_572_ CD8 + cytotoxic T cells; this immune response is associated with improved overall survival (OS) in patients with advanced/metastatic non-small cell lung cancer (NSCLC).

**Methods:**

A randomised, double blind, phase 2b trial, in HLA-A*201-positive patients with metastatic, TERT-expressing NSCLC, who did not progress after first-line platinum-based chemotherapy were randomised to receive either Vx-001 or placebo. The primary endpoint of the trial was OS.

**Results:**

Two hundred and twenty-one patients were randomised and 190 (101 and 89 patients in the placebo and the Vx-001 arm, respectively) were analysed for efficacy. There was not treatment-related toxicity >grade 2. The study did not meet its primary endpoint (median OS 11.3 and 14.3 months for the placebo and the Vx-001, respectively; *p* = 0.86) whereas the median Time to Treatment Failure (TTF) was 3.5 and 3.6 months, respectively. Disease control for >6months was observed in 30 (33.7%) and 26 (25.7%) patients treated with Vx-001 and placebo, respectively. There was no documented objective CR or PR. Long lasting TERT-specific immune response was observed in 29.2% of vaccinated patients who experienced a significantly longer OS compared to non-responders (21.3 and 13.4 months, respectively; *p* = 0.004).

**Conclusion:**

Vx-001 could induce specific CD8^+^ immune response but failed to meet its primary endpoint. Subsequent studies have to be focused on the identification and treatment of subgroups of patients able to mount an effective immunological response to Vx-001.

**Clinical trial registration:**

NCT01935154

## Background

The recent significant progress in immuno-oncology is based on the use of immune check-point inhibitors, which can break the local intratumoural immunosuppression by blocking the immune response inhibition pathways mediated by the PD-1/PD-L1 interaction.^1^ Immune check-point inhibitors are mainly effective in patients with immunogenic tumours, such as tumours with an important infiltration by tumour specific T cells (Tumour Infiltrating Lymphocytes; TILs). In contrast, they are not active in patients with non-immunogenic tumours that are weakly or not at all infiltrated by TILs.^2,3^ Tumour immunogenicity is closely related to the neoantigens, generated by gene mutations during oncogenesis and represented by the Tumor Mutational Burden (TMB); indeed, tumours with high TMB have been shown to be the most immunogenic and the most sensitive to immune check-point inhibitors.^4,5^ One of the key questions in immuno-oncology is, therefore, how to turn non-immunogenic tumours into immunogenic. One way to reach this objective is to use cancer vaccines which, by inducing a tumour specific immune response, will encompass the absence of an endogenous immunity.

All cancer vaccines, except Provenge in prostate cancer, tested in phase 3 trials, have been shown to be clinically inefficient. The common characteristic of all these vaccines is that they targeted tumour-associated antigens (TAA) that are expressed by both tumour and normal cells and are, therefore, involved in the self-tolerance process. As a consequence, immune system was not able to mount a strong immune response against TAA. This is not the case for the neoantigens, which represent a new family of antigens targeted by tumour vaccines.^6,7^ Neoantigens are recognised by the immune system as “non-self” and can trigger strong anti-tumour immunity. The main disadvantage of neoantigens is that they are very often patient-specific, must be identified for each patient individually and their use is limited to the patient they are identified from.

We have described a new family of tumour antigens named “optimized cryptic peptides”, which derive from universal tumour antigens and are recognised by the immune system as “non-self” being thus strongly immunogenic. It is noteworthy that several identified “neo-antigens” are naturally occurring optimised cryptic peptides.^8,9^ Vx-001 is the first vaccine based on the “optimized cryptic peptides” approach. Vx-001 targets TERT (TElomerase Reverse Transcriptase) and has been tested in a basket phase 1/2 clinical study on patients with different tumour types, mainly non-small cell lung cancer (NSCLC) and breast cancer; Vx-001 has been shown to be strongly immunogenic, with some objective clinical responses and significantly better overall survival in patients who developed a vaccine-specific immune response and, especially, in patients with pre-treated metastatic NSCLC.^10–14^

Based on these results, a multicentre, placebo-controlled, double blind, randomised phase 2b trial of Vx-001 was conducted in HLA-A*201-positive patients with metastatic and TERT-expressing NSCLC, who did not progress after 1st line platinum-based chemotherapy; the final results of the trial are presented in the current report.

## Methods

### Study design

Vx-001-201 is a multicentre, double blind, placebo controlled, randomised phase 2b study conducted in 70 sites in Europe. The study aimed to examine the role of the Vx-001 vaccine as maintenance immunotherapy in NSCLC patients who experienced disease control after 1st line chemotherapy (Supplementary Fig. S1). The protocol was approved by the institutional review boards and independent ethics committees of the participating centres and registered under the NCT01935154 identifier at the *clinicaltrials.gov* website. The study was performed in accordance with the Declaration of Helsinki and was conducted in compliance with the International Conference on Harmonization on Good Clinical Practice and written informed consent was required from all patients prior to enrolment. The study was sponsored and the investigational drug was provided free of charge by Vaxon-Biotech.

### Patients

Patients aged >18 years old, with histologically confirmed, metastatic NSCLC, an Eastern Cooperative Oncology Group (ECOG) performance status (PS) of 0–1 who experienced disease control [complete response (CR), partial response (PR) or stable disease (SD)] after four cycles of platinum-based front-line chemotherapy were eligible for this study. Additional key eligibility criteria included an HLA-A*0201 haplotype and tumoural expression of TERT as assessed by in situ hybridization in a central laboratory (Vaxon Biotech, Genopole, Evry, France), adequate marrow, renal and liver function tests. Patients were excluded if they had brain metastases, autoimmune or immunodeficiency diseases.

### Treatment plan

Patients enrolled in the study received the first vaccination within 4 weeks from the end of the fourth cycle of first-line chemotherapy. Patients were randomly assigned (1:1) to receive Vx-001 or placebo. Randomization was performed via an Interactive Web Response System (IWRS). Randomization was stratified according to the stage (stage IV *vs* recurrent stage I–III), the histology (non-squamous *vs* squamous) and the response to front-line chemotherapy (CR and PR vs SD).

Vx-001 is composed of two 9-amino acid-peptides, the optimised Vx-001/TERT_572Y_ and the wild-type (WT) Vx-001/TERT_572_. The Active Pharmaceutical Ingredients were produced by Bachem SA (Bubendorff, Switzerland) and the final products were manufactured by Amatsi (Saint Gély, France). Two placebos corresponding to each peptide solvents (with pH5.5 and pH3.5) were manufactured by Amatsi. Peptides and placebos were administered subcutaneously emulsified with the adjuvant Montanide ISA51VG (Seppic, Castres, France). Patients received six vaccinations cycles every 3 weeks. The first and the second vaccinations were done with the Vx-001/TERT_572Y_ (2 mg) while the following four vaccinations were done with the WT Vx-001/TERT_572_ (2 mg) peptide. Patients who continued controlling their disease after the sixth vaccination received boost vaccinations with the Vx-001/TERT_572_ (2 mg) every 3 months.

Post-chemotherapy disease control before treatment was documented by physical examination, complete blood cell count (CBC) with differential and platelet count and normal biochemical tests, electrocardiogram (ECG), computed tomography scans of the chest, abdomen and brain and whole-body bone scan. During treatment, physical examination, renal, hepatic and hematologic function tests were assessed after the third and sixth vaccination and thereafter after every boost vaccination.

Patients stopped vaccination because of disease progression according to investigator evaluation, toxicity, consent withdrawal or death. Post-vaccination treatment was at the discretion of the responsible physician.

### Clinical outcomes

The primary objective was a time-to-event comparison of overall survival (OS) in Vx-001 treated vs placebo-treated patients. The secondary objectives were the comparison of (i) survival rate at 12 months and (ii) Time to Treatment Failure (TTF) in Vx-001 treated vs placebo-treated patients. Additional exploratory objectives were the comparison of: (i) OS in immune responders versus non-responders to Vx-001; (ii) OS according to the presence of an hTERT specific immune response before the administration of Vx-001 vaccine and (iii) OS according to the high or low levels of TERT expression on the primary tumour.

### Statistical analysis

Statistical analysis was described in the pre-defined Statistical Analysis Plan (SAP) finalised before the placebo codes were broken (Supplementary Fig. S1). Given that the OS in the placebo group was assumed to be 9.8 months,^15–17^ an OS of 13.2 months (a 35.5% increase) was expected in the Vx-001 group, for an accrual period of 2 years and a minimum patients’ follow-up period of 6 months. Based on the above assumptions, 100 patients had to be enrolled in each arm in order to detect the pre-specified difference between the treatment arms with 82.5% power and a type I error of 5% (one-sided) significance level (Supplementary Fig. S1).

The primary analysis was based on the comparison of OS between the two arms using the Kaplan-Meier method and the Log-rank test. Full Analysis Set (FAS) (primary analysis set) was composed of all patients who were randomised, excluding patients who did not receive at least one dose of investigational product or placebo and all patients who violated major entry criteria. The independent effect of treatment as well as of different prognostic factors on OS were investigated using the Cox’s proportional hazards model. All *p* values <0.05 were considered statistically significant. All clinical data were held centrally and analysed using the SPSS (version 22.0) program. Final data update was performed on March 2017.

### Immunomonitoring

Vaccine-induced immune response was evaluated before the first vaccination (baseline), before the third vaccination (W6) and 3 weeks after the sixth vaccination (W18) or at the end of treatment visit in patients dropped out before the sixth vaccination. In boosted patients it was evaluated every 24 weeks.

Peripheral Blood Mononuclear Cells (PBMCs) were isolated and stored at −180 °C in a central laboratory (Texcell, Evry, France). All samples were tested at the end of the study using the IFNγ ELISpot assay. IFNγ ELISpot assay was performed using the Human IFNγ ELISpot PVDF-Enzymatic kit (Diaclone, Besançon, France) and analysed with an automated reader AID (Germany) as has been previously reported.^13^ In brief, PBMCs (2 × 10^5^) were incubated in the presence of 10 µM of an irrelevant peptide as negative control (group A), Vx-001/TERT_572Y_ peptide (group B), Vx-001/TERT_572_ peptide (group C) and 2 µg/ml of CEF (immunogenic peptides from Cytomegalovirus, Epstein-Barr virus and Flu virus) peptide pool (group D) and 2.5 µg/ml PHA (group E) as positive controls. Results were expressed as mean number of specific T cells (spots)±SD/10^6^ PBMCs. Statistical analysis for positivity was done using the Student *t*-test (*p* < 0.05) between groups A and B, A and C, A and D and A and E. ELISpot assay was considered evaluable when there was a significant difference between groups A and D. A group (B, C or D) was considered positive when there was a: (i) statistically significant difference (*p* < 0.05) between this group and group A and (ii) difference of more than 10 spots between this group and group A.

The number of spots producing specific T cells was calculated in ELISpot positive B or C groups according to the formula:

#### Average number of spots in the B or C groups–average number of spots in group A

All patients who developed an immune response against the TERT_572_ peptide were considered as “immune responders” to Vx-001. For patients with pre-existing immune response to TERT_572_ (natural immunity) immune responders to Vx-001 should increase the frequency of TERT_572_ specific CD8 + cells by at least two folds.^11–14^ All criteria for immune response and immune responders were pre-defined before the placebo codes were broken and before the first patient was tested.

## Results

### Patients

Between August 2012 and March 2016, 1407 patients were screened and 221 were randomised (Supplementary Fig. S1 shows the consort flow diagram). The main reason of non-eligibility was HLA-A*0201 negativity (788 patients). Thus, 221 patients were considered eligible for the study and 112 patients were randomised to receive placebo and 109 patients Vx-001. Thirty-one patients were excluded from the FAS (17 and 14 patients were excluded before and after the placebo code was broken, respectively) because of major violation of inclusion/exclusion criteria as pre-defined in the Statistical Analysis Plan (25 patients entered the study with progressive disease, five did not have metastatic/recurrent NSCLC and one had a SCLC). Therefore, final analysis was performed for 190 patients (89 in the Vx-001 arm and 101 in the placebo arm (Supplementary Fig. S1).

There were 132 (69.5%) males, 47.9% of the patients were <65years old, 13.2% were never smokers, 60.5% had a PS of ECOG 1, and 60% of them  had a non-squamous cell histology; moreover, 46.3% of patients had documented objective response (complete or partial response) at the time of study entry. Patients’ characteristics were well balanced across the two treatment arms (Table 1).

**Table 1:** Patients characteristics.

	All		Vx-001		Placebo	
	No of patients	%	No of patients	%	No of patients	%
	190		89		101	
Sex						
Males	132	69.5	60	67.4	72	71.3
Females	58	30.5	29	32.6	29	28.7
Age						
>65 years	99	52.1	45	50.6	54	53.5
<65 years	91	47.9	44	49.4	47	46.5
Histology						
NSQ	114	60.0	55	61.8	59	58.4
SQ	72	37.9	34	38.2	38	37,6
Mixte	4	2.1	0	0.0	4	4,0
Response to previous treatment						
OR	88	46.3	36	40.4	52	51.5
SD	102	53.7	53	59.6	49	48.5
ECOG						
0	75	39.5	33	37.1	42	41.6
1	115	60.5	56	62.9	59	58.4
Smoking status						
Never	25	13.2	10	11.2	15	14.9
Smokers	165	86.8	79	88.8	86	85.1
Heavy smokers (>25 years)	128	67.4	62	69.7	66	65.3
Light smokers (<25 years)	35	18.4	17	19.1	18	17.8
Missing	2	1.1	0	0,0	2	2.0

### Treatment and adverse events

A total of 43 (48.3%) patients enrolled in the Vx-001 arm and 45 (44.55%) patients enrolled in the placebo arm completed the six vaccination cycles (*p* = 0.66); moreover, 18 (17.8%) and 25 (28.08%) patients enrolled in the placebo and the Vx-001 arm, respectively (*p* = 0.12) received boost vaccinations. In addition, 139 patients received subsequent cancer treatment after disease progression and 30 of them immune check-point inhibitors (16 in the placebo arm and 14 in the Vx-001 arm). There was no patient requiring treatment discontinuation because of severe grade 3 or 4 adverse events.

The tolerance of the treatment was excellent since Vx-001-related adverse events were mainly Grade 1 and Grade 2 local reactions at the site of injection (*n* = 14). These local reactions were mainly attributed to the used adjuvant (Montanide ISA51 VG). Moreover, a patient developed Grade 3 fever, which completely resolved with paracetamol for 2 days.

### Monitoring of immune response to TERT_572_

TERT_572_-specific immune response induced by Vx-001 was evaluated in 75 patients treated with Vx-001 and 79 patients who received placebo using ex vivo IFNγ ELISpot assay. Twenty-two Vx-001-treated patients (29.3%) developed a TERT_572_ specific immune response detected at W6 and/or W18. This Vx-001 induced response was maintained by boost vaccinations in four patients whereas no patient in the placebo arm had detectable TERT_572_-specific immune response at W18 (data not shown). Pre-vaccine baseline TERT_572_ immune response (natural immunity) was evaluated in 167 patients (87 placebo and 80 Vx-001) and was detected in 45 patients (27%) (placebo arm: *n* = 21; Vx-001 arm: *n* = 24). Vx-001 induced an immune response more frequently in patients without natural immunity (36.2%) than in patients with natural immunity (15%); Vx-001 amplified natural immunity only in one patient at W6.

### Response to treatment

There was no documented objective CR or PR according to the RESIST criteria. Forty-four (43.6%) and 42 (47.2%) patients in the placebo and the Vx-001 arm, respectively, experienced disease control after 3 treatment cycles (2 or 3 months). The remaining patients in both-groups experienced disease progression within the first 3 months of treatment. In addition, 30 (33.7%) and 26 (25.7%) patients treated with Vx-001 and placebo, respectively, experienced disease control for more than 6 months, (*p* = 0.26).

### Overall survival and time to treatment failure

**Fig. 1:**
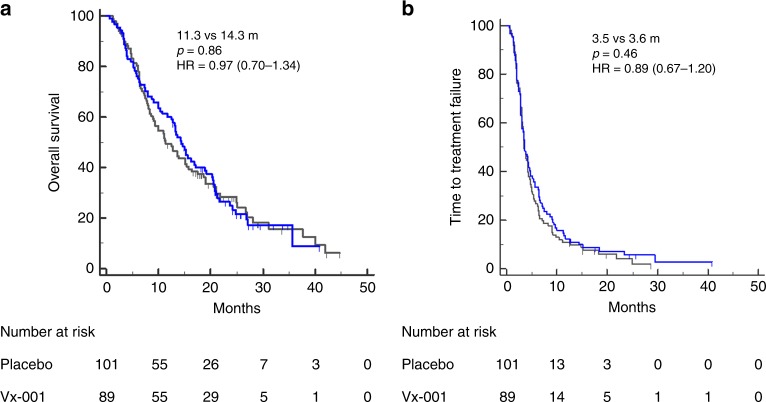
OS and TTF in the Full Analysis Set (FAS) population. Comparison of OS **a** and TTF **b** between placebo-treated (black line) and Vx-001 treated (blue line) patients.

**Fig. 2:**
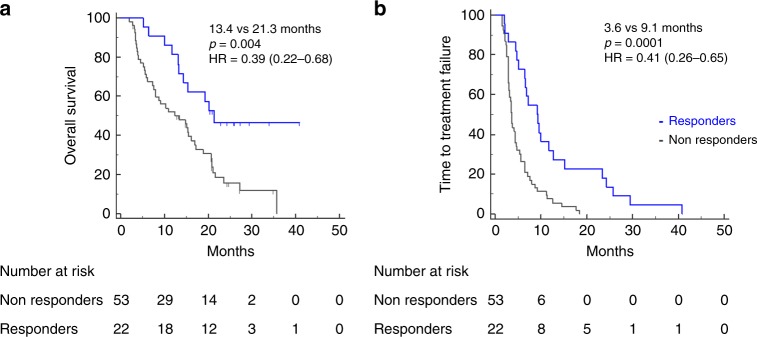
Correlation between Vx-001-induced immune response and clinical response. OS **a** and TTF **b** in Vx-001 immune responders (blue line) and non-responders (black line).

After a median follow-up period of 13.6 and 11.3 months in the Vx-001 and placebo arm, respectively, 84 (94.4%) and 96 (95%) patients had experienced disease progression (*p* = 0.99); moreover, 68 (76.4%) and 79 (78.2%) deaths occurred in the Vx-001 and the placebo group, respectively (*p* = 0.86). The median OS was 14.3 months and 11.3 months in the Vx-001- and the placebo-treated patients, respectively (HR = 0.96, *p* = 0.86; Fig. 1a). The 1-yr OS was 58.4% and 48.5% for the Vx-001 and the placebo group, respectively (*p* = 0.19). The median TTF was 3.5 and 3.6 months in patients enrolled in the Vx-001 and placebo arm, respectively (HR = 0.89; *p* = 0.86; Fig. 1b). Vx-001-triggered immune response was correlated with a better clinical outcome. The median OS of the Vx-001 immune responders was 21.3 months compared to 13.4 months for immune non-responders (HR:0.39, *p* = 0.004; Fig. 2a). Similarly, the median TTF was significantly longer in immune responders than in immune non-responders (9.1 vs 3.6 months, respectively: HR: 0. 41; *p* = 0.0001) (Fig. 2b). Since the increased serum levels of LDH and γGT represent poor prognostic factors in NSCLC,^18–21^ an additional unplanned and not pre-specified exploratory analysis was performed, showing that in vaccinated patients with high LDH/γGT serum level both the median OS and median TTF were significantly higher compared to placebo-treated patients (OS: 16.2 months versus 9.3 months; HR = 0.50, *p* = 0.003 and TTF: 4.5 months versus 3.3 months: HR = 0.54; *p* = 0.0019).

## Discussion

The current phase 2 multicentre, randomised, placebo-controlled Phase 2b trial of the Vx-001 vaccine, which was conducted in HLA-A*0201 patients with TERT-expressing metastatic NSCLC who did not progress after 1st line platinum-based chemotherapy, failed to meet its primary endpoint by demonstrating a survival benefit in the Vx-001-treated patients compared to those who received the placebo.

Immunomonitoring revealed that almost 29% of Vx-001 treated patients mounted a TERT_572_ specific immune response. This confirms previous results obtained in the phase 1/2 study conducted by our group.^11^ It is noteworthy that this immune response was evaluated ex vivo and did not need any in vitro amplification to be detected. Ex vivo detection of vaccine-induced immune response was reported in only 10 out of 58 vaccination studies which have been reviewed (Supplementary Table S1). In all these 58 studies, vaccines targeted dominant peptides from TAA and failed to stimulate a strong T cell response because of the self-tolerance. Surprisingly, the need of in vitro amplification to detect a vaccine-induced immune response was also observed in two clinical trials with neoantigens.^6,7^ An interesting observation of the current study was that 27% of patients showed a pre-vaccine immunity against TERT_572_ (natural immunity). Vx-001 amplified the TERT_572_ specific T cells in only one out of 24 patients with natural immunity. This finding also confirms previous results of the phase 1/2 trial^11^ as well as from other studies where amplification of pre-existing immune response, measured by a functional test such as production of cytokines, is a rather rare phenomenon (Supplementary Table S2).

Despite the fact that Vx-001 failed to demonstrate a survival benefit in the entire group of vaccinated patients, immune responders experienced a statistically significant improvement of OS and TTF compared to patients who failed to respond to Vx-001 vaccine. In addition, an unplanned subgroup analysis revealed that vaccination with Vx-001 was associated with a better clinical outcome, both in terms of OS and TTF, in patients with increased serum levels of LDH and γGT, which define a subgroup of patients with an unfavourable prognosis.^18–21^ Interestingly, serum LDH is a predictive marker of efficacy of Immune Check-Point Inhibitors in NSCLC and melanoma.^18^ Moreover, it is noteworthy that normal LDH and γGT levels were observed more frequently in patients without natural immunity (67%) who responded to Vx-001 than in patients with natural immunity (42%) who experienced a poor immune response to Vx-001 (*p* = 0.03).

These observations further confirm our previous studies with the Vx-001 vaccine in pre-treated NSCLC^14^ patients and suggest that Vx-001 could provide clinical benefit, at least, in some specific subgroup(s) of patients. However, we cannot exclude that immune responders constitute a subgroup of patients that would have survived longer even without vaccine. It should be noted that a correlation between clinical response and vaccine-induced immune response is not a common finding in studies with cancer vaccines; such a correlation has been observed with a Survivin vaccine in melanoma,^22^ the GV1001 in stage IV NSCLC^23^ whereas it was not reported for NeuVax vaccine in breast cancer,^24^ the IMA901 in RCC,^25^ the PROSTVAC-VF in prostate cancer,^26^ the STn-KLH in breast cancer,^27^ and the Stimuvax (BLP25) in NSCLC.^28^ In addition, in vaccine clinical trials in melanoma, immune response was not correlated with clinical benefit.^29^ Finally, a meta-analysis of 38 clinical trials could not demonstrate a clear correlation between immune response and objective clinical response.^30^ One probable reason for this observation would be the failure of the used vaccines to trigger a strong enough immune response, which would be translated to objective clinical response since these vaccines corresponded to dominant peptides of TAA, which are not strong immunogenic.

In conclusion, the current study although failed to meet its primary endpoint provides clear evidence that the Vx-001 vaccine may confer survival benefit in NSCLC patients who can mount an immune response upon vaccination with this vaccine. Further studies are required in order to define the subgroup(s) of patients who have the greater probability to respond to Vx-001 allowing, thus, the possibility to conduct a prospective trial to this selected population.

## Supplementary information

Supplementary_Tables

Suppl.Fig.S1

Statistical Analysis Plan

## Data Availability

All data are available via the corresponding author and the NCT trial centre.
