# Supplementary material for: Clinical activity of a htert (vx-001) cancer vaccine as post-chemotherapy maintenance immunotherapy in patients with stage IV non-small cell lung cancer: final results of a randomised phase 2 clinical trial
Source: Br J Cancer. 2020 Mar 25;122(10):1461–6. doi: 10.1038/s41416-020-0785-y (PMC7217860; doi:10.1038/s41416-020-0785-y)
Supplement: Supplementary file 1 — Supplementary_Tables [file 41416_2020_785_MOESM1_ESM.docx]

# Supplementary tables

## **Suppl. Table S1 :** Comparison of immune response in Vx001-201 study and other mmunotherapeutic vaccine based clinical trials.

| **Cancer** | **Target** | **Adjuvant** | **Patients** | **Comments** | **Ref.** |
| --- | --- | --- | --- | --- | --- |
| Melanoma | MAGE, MelanA, gp100, tyrosinase | DC | 14 | In vitro expansion | [[1](#_ENREF_1)] |
| Melanoma | MelanA, tyrosinase, MAGE | DC, KLH | 18 | In vitro expansion | [[2](#_ENREF_2)] |
| Melanoma | gp100 | Montanide | 14 | In vitro expansion | [[3](#_ENREF_3)] |
| Melanoma | Gp100, tyrosinase | Montanide GM-CSF | 42 | In vitro expansion | [[4](#_ENREF_4)] |
| Melanoma | Gp100, tyrosinase | Montanide GM-CSF | 26 | In vitro expansion | [[5](#_ENREF_5)] |
| Melanoma | MelanA, gp100 | Montanide | 41 | Ex vivo | [[6](#_ENREF_6)] |
| Melanoma | MelanA | Montanide | 20 | In vitro expansion | **[**[**7**](#_ENREF_7)**]** |
| Breast | p53 | DC | 6 | In vitro expansion | [[8](#_ENREF_8)] |
| Various | Survivin (multiple peptides) | Montanide | 49 | Ex vivo | [[9](#_ENREF_9)] |
| Various | Survivin (multiple peptides) | Montanide | 18 | In vitro expansion | [[9](#_ENREF_9)] |
| Breast | E75 | Montanide, GM-CSF | 13 | In vitro expansion | [[10](#_ENREF_10)] |
| Ovarian | Multi HER2 | Montanide, GM-CSF | 9 | In vitro expansion | [[11](#_ENREF_11)] |
| Ovarian | p53 (long peptide) | Montanide | 20 | In vitro expansion | [[12](#_ENREF_12)] |
| mCRC | p53 (long peptide) | Montanide | 9 | In vitro expansion | [[13](#_ENREF_13)] |
| prostate | PSA | Montanide | 5 | In vitro expansion | [[14](#_ENREF_14)] |
| Lung, meso | WT1 | Montanide, GM-CSF | 11 | In vitro expansion | [[15](#_ENREF_15)] |
| Gastric | HER-2 | DC | 9 | In vitro expansion | [[16](#_ENREF_16)] |
| Prostate, breast | TERT | DC | 7 | In vitro expansion | [[17](#_ENREF_17)] |
| Prostate | HER-2 | FLT-3 | 20 | In vitro expansion | [[18](#_ENREF_18)] |
| RCC | TERT | Montanide | 14 | In vitro expansion | [[19](#_ENREF_19)] |
| Various | K-ras, p53 | DC | 37 | In vitro expansion | [[20](#_ENREF_20)] |
| Prostate | HER-2 | Montanide, GM-CSF | 17 | In vitro expansion | [[21](#_ENREF_21)] |
| Pancreas | K-ras | Montanide, GM-CSF | 43 | In vitro expansion | [[22](#_ENREF_22)] |
| HCC | TERT | Montanide | 14 | Ex vivo | [[23](#_ENREF_23)] |
| Ovarian | p53 | DC | 19 | Ex vivo | [[24](#_ENREF_24)] |
| H&N | p53 | DC | 16 | Ex vivo | [[25](#_ENREF_25)] |
| Prostate | TARP | Montanide, GM-CSF | 41 | In vitro expansion | [[26](#_ENREF_26)] |
| Various | NY-ESO1 | montanide | 28 | In vitro expansion | [[27](#_ENREF_27)] |
| Ovarian | NY-ESO1 | CpG | 9 | In vitro expansion | [[28](#_ENREF_28)] |
| Various | NY-ESO1 | Montanide | 10 | In vitro expansion | [[29](#_ENREF_29)] |
| Various | NY-ESO1 | Montanide | 20 | In vitro expansion | [[30](#_ENREF_30)] |
| NSCLC | OSE-2101 | Montanide | 64 | In vitro expansion | [[31](#_ENREF_31)] |
| Cholangio | multipeptide | Montanide | 9 | In vitro expansion | [[32](#_ENREF_32)] |
| NSCLC | multipeptide | Montanide | 15 | In vitro expansion | [[33](#_ENREF_33)] |
| Oesophagous | multipeptide | Montanide | 10 | In vitro expansion | [[34](#_ENREF_34)] |
| Oesophagous | multipeptide | Monanide | 60 | In vitro expansion | [[35](#_ENREF_35)] |
| Cervical | HPV peptides | Montanide | 6 | In vitro expansion | [[36](#_ENREF_36)] |
| Multiple Myeloma | MUC-1 | BLP | 34 | In vitro expansion | [[37](#_ENREF_37)] |
| Prostate | NY-ESO-1 | Montanide | 14 | In vitro expansion | [[38](#_ENREF_38)] |
| Brain | multipeptide | Montanide | 26 | In vitro expansion | [[39](#_ENREF_39)] |
| H&N | multipeptide | Montanide | 21 | In vitro expansion | [[40](#_ENREF_40)] |
| Prostate | lysates | DC | 14 | In vitro expansion | [[41](#_ENREF_41)] |
| mUC | survivin | Montanide | 21 | In vitro expansion | [[42](#_ENREF_42)] |
| mCRC | multipeptide | Montanide | 18 | In vitro expansion | [[43](#_ENREF_43)] |
| mCRC | survivin | Montanide | 5 | In vitro expansion | [[44](#_ENREF_44)] |
| Solid tumours | K-ras | GM-CSF | 21 | In vitro expansion | [[45](#_ENREF_45)] |
| mRCC | IMA-9101 |  | 98 | In vitro expansion | [[46](#_ENREF_46)] |
| NSCLC | IDO5 | Montanide | 15 | In vitro expansion | [[47](#_ENREF_47)] |
| GBM | multipeptide | Montanide | 23 | In vitro expansion | [[48](#_ENREF_48)] |
| melanoma | neoantigen | Montanide | 10 | In vitro expansion | [[49](#_ENREF_49)] |
| melanoma | AutologousmRNA |  | 3 | In vitro expansion | [[50](#_ENREF_50)] |
| melanoma | Neoantigen-mRNA |  | 19 | In vitro expansion | [[51](#_ENREF_51)] |
| melanoma | lysates | DC | 40 | In vitro expansion | [[52](#_ENREF_52)] |
| melanoma | TERT, survivin, p53 | Montanide | 28 | In vitro expansion | [[53](#_ENREF_53)] |
| mCRC | p53 | Montanide | 11 | Ex vivo | [[54](#_ENREF_54)] |
| mCRC | CEA | ALVAC | 118 | Ex vivo | [[55](#_ENREF_55)] |
| Prostate | PARP-DNA |  | 16 | Ex vivo | [[56](#_ENREF_56)] |
| Various | lysate | DC | 24 | Ex vivo | [[57](#_ENREF_57)] |
| prostate | PROSTVAC |  | 10 | Ex vivo | [[58](#_ENREF_58)] |

## **Suppl. Table S2:** Amplification of pre-existing immune response in other vaccine based clinical trials

| **Cancer** | **Target** | **Pre-existingresponse (n=99)** | **Notamplified (n=85)** | **Amplified (n=14)** | **Ref.** |
| --- | --- | --- | --- | --- | --- |
| HCC | TERT | 11 | 10 | 1 | [[23](#_ENREF_23)] |
| Breast | E75 | 5 | 5 | 0 | [[10](#_ENREF_10)] |
| mCRC | p53 (long peptide) | 2 | 2 | 0 | [[13](#_ENREF_13)] |
| Ovarian | NY-ESO1 | 2 | 2 | 0 | [[28](#_ENREF_28)] |
| various | NY-ESO1 | 6 | 5 | 1 | [[27](#_ENREF_27)] |
| Melanoma | MelanA, gp100 | 23 | 20 | 3 | [[7](#_ENREF_7)] |
| Ovarian | p53 (long peptide) | 3 | 1 | 2 | [[12](#_ENREF_12)] |
| various | NY-ESO1 | 4 | 3 | 1 | [[30](#_ENREF_30)] |
| Pancreas | KIF20A | 4 | 2 | 2 | [[59](#_ENREF_59)] |
| mCRC | p53 | 2 | 2 | 0 | [[54](#_ENREF_54)] |
| Prostate | PSA | 4 | 4 | 0 | [[58](#_ENREF_58)] |
| melanoma | mRNA | 3 | 3 | 0 | [[50](#_ENREF_50)] |
| melanoma | TERT, survivin, p53 | 8 | 6 | 2 | [[53](#_ENREF_53)] |
| melanoma | lysates | 5 | 3 | 2 | [[52](#_ENREF_52)] |
| breast | HER-2 | 10 | 10 | 0 | [[60](#_ENREF_60)] |
| CRC, NSCLC | CEA | 1 | 1 | 0 | [[61](#_ENREF_61)] |
| pancreas | CEA, TERT, survivin | 6 | 6 | 0 | [[62](#_ENREF_62)] |

## **Suppl. Table S3:** Ethical approval and consent to participate

The protocol was approved by the institutional review boards and independent ethics committees of the participating centers:

| **Czechia** | |
| --- | --- |
| Fakultni nemocnice Plzen |  |
| Plzen-Bory, Czechia, 30599 | |
| Vseobecna fakultni nemocnice |  |
| Praha 2, Czechia, 18008 | |
| **France** | |
| University Hospital of Angers |  |
| Angers, France, 49933 | |
| Ambroise Paré Hospital |  |
| Boulogne Billancourt, France, 92104 | |
| Hopital Nord |  |
| Marseille, France, 13915 | |
| Pitié-Salpetrière Hospital |  |
| Paris, France, 75651 | |
| Pontchaillou Hospital |  |
| Rennes, France, 35033 | |
| **Germany** | |
| Hospital Grosshansdorf |  |
| Grosshansdorf, Germany, 22927 | |
| Lungenklinik Hemer |  |
| Hemer, Germany, 58675 | |
| Klinikum Kassel GmbH |  |
| Kassel, Germany, 34125 | |
| University Medical center Kiel |  |
| Kiel, Germany, 24116 | |
| Klinik Löwenstein |  |
| Löwenstein, Germany, 74245 | |
| Chirurgische Klinik-Inderdisziplinäre Thorakale Oncologie |  |
| Mannheim, Germany, 68167 | |
| Mühlenkreiskliniken (AöR) Johannes Wesling Klinikum Minden |  |
| Minden, Germany, 32429 | |
| LMU Klinikum der Universität München |  |
| Munich, Germany, 80336 | |
| Pius Hospital Oldenburg |  |
| Oldenburg, Germany, 26121 | |
| Mathias Spital Rheine Medizinische Klinik V |  |
| Rheine, Germany, 48431 | |
| **Greece** | |
| University Hospital of Alexandroupolis |  |
| Alexandroupolis, Greece, 68100 | |
| 251 General Airforce Hospital |  |
| Athens, Greece, 11525 | |
| General hospital of thoracic diseases Sotiria |  |
| Athens, Greece, 11527 | |
| Aretaieio Hospital |  |
| Athens, Greece, 11528 | |
| General Hospital Alexandra |  |
| Athens, Greece, 11528 | |
| Iaso General Hospital Cholargos |  |
| Athens, Greece, 15562 | |
| University Hospital of Heraklion |  |
| Heraklion, Greece, 71110 | |
| University General Hospital of Ioannina |  |
| Ioannina, Greece, 45500 | |
| General Oncology Hospital of Kifissia Agioi Anargyroi |  |
| Nea Kifissia, Greece, 14564 | |
| University Hospital of Patras |  |
| Rio, Greece, 26504 | |
| University General Hospital Papageorgiou |  |
| Thessaloniki, Greece, 56429 | |
| General Hospital G. Papanikolaou |  |
| Thessaloniki, Greece, 57010 | |
| **Italy** | |
| SG Moscati Hospital |  |
| Avellino, Italy, 83100 | |
| G. Rummo Hospital |  |
| Benevento, Italy, 82100 | |
| Mater Domini Catanzaro Hospital |  |
| Catanzaro, Italy, 88100 | |
| San Paolo Hospital |  |
| Milano, Italy, 20142 | |
| European Institute of Oncology |  |
| Milan, Italy, 20141 | |
| Seconda Universita degli Studi Napoli Hospital |  |
| Naples, Italy, 80131 | |
| Insituto Oncologico Veneto |  |
| Padova, Italy, 35128 | |
| S. Maria della Misericordia Hospital |  |
| Perugia, Italy, 06132 | |
| A.O.U di Pisa Hospital |  |
| Pisa, Italy, 56126 | |
| S. Maria Nuova Hospital |  |
| Reggio Emilia, Italy, 42100 | |
| University Hospital of Siena |  |
| Siena, Italy, 53100 | |
| **Poland** | |
| Uniwersytet Medyczny Bialistok |  |
| Bialystok, Poland, 15540 | |
| Hospicjum im. ks. T. Dutkiewicza SAC |  |
| Gdansk, Poland, 80208 | |
| Przychodnia KOMED |  |
| Konin, Poland, 62500 | |
| Nzoz Vesalius |  |
| Krakow, Poland, 31108 | |
| MS Clinsearch |  |
| Lublin, Poland, 20064 | |
| Oddział Onkologii z Pododdziałem Chemioterapii Nowotworów Płuc |  |
| Olsztyn, Poland, 10357 | |
| Mazowieckie Centrum Leczenia Chorób Płuc i Gruźlicy w Otwocku |  |
| Otwock, Poland, 05400 | |
| Med-Polonia |  |
| Poznan, Poland, 60693 | |
| Specjalistyczny Szpital im. prof.Alfreda Sokołowskiego |  |
| Szczecin, Poland, 70891 | |
| Centrum Onkologii - Instytut im. M. Skłodowskiej - Curie |  |
| Warszawa, Poland, 02781 | |
| Fundacja Hospicjum Onkologiczne św. Krzysztofa |  |
| Warszawa, Poland, 02781 | |
| **Romania** | |
| SC Oncopremium Team SRL |  |
| Baia Mare, Romania | |
| Spit. Jud. de Urgenţă |  |
| Brăila, Romania | |
| Spitalul Universitar de Urgenta Bucuresti |  |
| Bucuresti, Romania, 50098 | |
| Institutului Oncologic "Prof. Dr. I. Chiricuţă" Cluj-Napoca |  |
| Cluj-Napoca, Romania, 400015 | |
| Medisprof SRL |  |
| Cluj-Napoca, Romania, 400058 | |
| Oncolab SRL |  |
| Craiova, Romania, 200385 | |
| Spitalul Clinic Judetean de Urgenta Sibiu |  |
| Sibiu, Romania, 550245 | |
| Spitalul Clinic Judetean de Urgenta "Sf. Ioan cel Nou" |  |
| Suceava, Romania | |
| Oncomed SRL, Department of Medical Oncology |  |
| Timisoara, Romania | |
| **Spain** | |
| USP Institut Universitari Dexeus |  |
| Barcelona, Spain, 08028 | |
| University Hospital P. Vall d'Hebron |  |
| Barcelona, Spain, 08035 | |
| Hospital de la Santa Creu I Sant Pau |  |
| Barcelona, Spain, 08041 | |
| Ico-Hospital Germans Trias I Pujol |  |
| Barcelona, Spain, 08916 | |
| Hospital Provencial de Castellón |  |
| Castellón, Spain, 12002 | |
| Oncologia Medica FUNDACIÓN JIMÉNEZ DÍAZ |  |
| Madrid, Spain, 28040 | |
| Hospital Univesitario Puerta de Hierro |  |
| Madrid, Spain, 28222 | |
| H.R.U. Carlos Haya Málaga |  |
| Málaga, Spain, 29010 | |
| Hospital Son Llatzer |  |
| Palma de Mallorca, Spain | |
| Hospital Clinico de Valencia |  |
| Valencia, Spain, 46010 | |

# Bibliography

1. Mackensen A, Herbst B, Chen JL, Kohler G, Noppen C, Herr W, Spagnoli GC, Cerundolo V and Lindemann A. Phase I study in melanoma patients of a vaccine with peptide-pulsed dendritic cells generated in vitro from CD34(+) hematopoietic progenitor cells. International journal of cancer Journal international du cancer. 2000; 86(3):385-392.

2. Banchereau J, Palucka AK, Dhodapkar M, Burkeholder S, Taquet N, Rolland A, Taquet S, Coquery S, Wittkowski KM, Bhardwaj N, Pineiro L, Steinman R and Fay J. Immune and clinical responses in patients with metastatic melanoma to CD34(+) progenitor-derived dendritic cell vaccine. Cancer research. 2001; 61(17):6451-6458.

3. Slingluff CL, Jr., Yamshchikov G, Neese P, Galavotti H, Eastham S, Engelhard VH, Kittlesen D, Deacon D, Hibbitts S, Grosh WW, Petroni G, Cohen R, Wiernasz C, Patterson JW, Conway BP and Ross WG. Phase I trial of a melanoma vaccine with gp100(280-288) peptide and tetanus helper peptide in adjuvant: immunologic and clinical outcomes. Clinical cancer research : an official journal of the American Association for Cancer Research. 2001; 7(10):3012-3024.

4. Weber J, Sondak VK, Scotland R, Phillip R, Wang F, Rubio V, Stuge TB, Groshen SG, Gee C, Jeffery GG, Sian S and Lee PP. Granulocyte-macrophage-colony-stimulating factor added to a multipeptide vaccine for resected Stage II melanoma. Cancer. 2003; 97(1):186-200.

5. Slingluff CL, Jr., Petroni GR, Yamshchikov GV, Barnd DL, Eastham S, Galavotti H, Patterson JW, Deacon DH, Hibbitts S, Teates D, Neese PY, Grosh WW, Chianese-Bullock KA, Woodson EM, Wiernasz CJ, Merrill P, et al. Clinical and immunologic results of a randomized phase II trial of vaccination using four melanoma peptides either administered in granulocyte-macrophage colony-stimulating factor in adjuvant or pulsed on dendritic cells. Journal of clinical oncology : official journal of the American Society of Clinical Oncology. 2003; 21(21):4016-4026.

6. Phan GQ, Touloukian CE, Yang JC, Restifo NP, Sherry RM, Hwu P, Topalian SL, Schwartzentruber DJ, Seipp CA, Freezer LJ, Morton KE, Mavroukakis SA, White DE and Rosenberg SA. Immunization of patients with metastatic melanoma using both class I- and class II-restricted peptides from melanoma-associated antigens. Journal of immunotherapy. 2003; 26(4):349-356.

7. Wang F, Bade E, Kuniyoshi C, Spears L, Jeffery G, Marty V, Groshen S and Weber J. Phase I trial of a MART-1 peptide vaccine with incomplete Freund's adjuvant for resected high-risk melanoma. Clinical cancer research : an official journal of the American Association for Cancer Research. 1999; 5(10):2756-2765.

8. Svane IM, Pedersen AE, Johnsen HE, Nielsen D, Kamby C, Gaarsdal E, Nikolajsen K, Buus S and Claesson MH. Vaccination with p53-peptide-pulsed dendritic cells, of patients with advanced breast cancer: report from a phase I study. Cancer immunology, immunotherapy : CII. 2004; 53(7):633-641.

9. Lennerz V, Gross S, Gallerani E, Sessa C, Mach N, Boehm S, Hess D, von Boehmer L, Knuth A, Ochsenbein AF, Gnad-Vogt U, Zieschang J, Forssmann U, Woelfel T and Kaempgen E. Immunologic response to the survivin-derived multi-epitope vaccine EMD640744 in patients with advanced solid tumors. Cancer immunology, immunotherapy : CII. 2014; 63(4):381-394.

10. Patil R, Clifton GT, Holmes JP, Amin A, Carmichael MG, Gates JD, Benavides LH, Hueman MT, Ponniah S and Peoples GE. Clinical and immunologic responses of HLA-A3+ breast cancer patients vaccinated with the HER2/neu-derived peptide vaccine, E75, in a phase I/II clinical trial. Journal of the American College of Surgeons. 2010; 210(2):140-147.

11. Chianese-Bullock KA, Irvin WP, Jr., Petroni GR, Murphy C, Smolkin M, Olson WC, Coleman E, Boerner SA, Nail CJ, Neese PY, Yuan A, Hogan KT and Slingluff CL, Jr. A multipeptide vaccine is safe and elicits T-cell responses in participants with advanced stage ovarian cancer. Journal of immunotherapy. 2008; 31(4):420-430.

12. Leffers N, Lambeck AJ, Gooden MJ, Hoogeboom BN, Wolf R, Hamming IE, Hepkema BG, Willemse PH, Molmans BH, Hollema H, Drijfhout JW, Sluiter WJ, Valentijn AR, Fathers LM, Oostendorp J, van der Zee AG, et al. Immunization with a P53 synthetic long peptide vaccine induces P53-specific immune responses in ovarian cancer patients, a phase II trial. International journal of cancer Journal international du cancer. 2009; 125(9):2104-2113.

13. Speetjens FM, Kuppen PJ, Welters MJ, Essahsah F, Voet van den Brink AM, Lantrua MG, Valentijn AR, Oostendorp J, Fathers LM, Nijman HW, Drijfhout JW, van de Velde CJ, Melief CJ and van der Burg SH. Induction of p53-specific immunity by a p53 synthetic long peptide vaccine in patients treated for metastatic colorectal cancer. Clinical cancer research : an official journal of the American Association for Cancer Research. 2009; 15(3):1086-1095.

14. Kouiavskaia DV, Berard CA, Datena E, Hussain A, Dawson N, Klyushnenkova EN and Alexander RB. Vaccination with agonist peptide PSA: 154-163 (155L) derived from prostate specific antigen induced CD8 T-cell response to the native peptide PSA: 154-163 but failed to induce the reactivity against tumor targets expressing PSA: a phase 2 study in patients with recurrent prostate cancer. Journal of immunotherapy. 2009; 32(6):655-666.

15. Krug LM, Dao T, Brown AB, Maslak P, Travis W, Bekele S, Korontsvit T, Zakhaleva V, Wolchok J, Yuan J, Li H, Tyson L and Scheinberg DA. WT1 peptide vaccinations induce CD4 and CD8 T cell immune responses in patients with mesothelioma and non-small cell lung cancer. Cancer immunology, immunotherapy : CII. 2010; 59(10):1467-1479.

16. Kono K, Takahashi A, Sugai H, Fujii H, Choudhury AR, Kiessling R and Matsumoto Y. Dendritic cells pulsed with HER-2/neu-derived peptides can induce specific T-cell responses in patients with gastric cancer. Clinical cancer research : an official journal of the American Association for Cancer Research. 2002; 8(11):3394-3400.

17. Vonderheide RH, Domchek SM, Schultze JL, George DJ, Hoar KM, Chen DY, Stephans KF, Masutomi K, Loda M, Xia Z, Anderson KS, Hahn WC and Nadler LM. Vaccination of cancer patients against telomerase induces functional antitumor CD8+ T lymphocytes. Clinical cancer research : an official journal of the American Association for Cancer Research. 2004; 10(3):828-839.

18. McNeel DG, Knutson KL, Schiffman K, Davis DR, Caron D and Disis ML. Pilot study of an HLA-A2 peptide vaccine using flt3 ligand as a systemic vaccine adjuvant. Journal of clinical immunology. 2003; 23(1):62-72.

19. Parkhurst MR, Riley JP, Igarashi T, Li Y, Robbins PF and Rosenberg SA. Immunization of patients with the hTERT:540-548 peptide induces peptide-reactive T lymphocytes that do not recognize tumors endogenously expressing telomerase. Clinical cancer research : an official journal of the American Association for Cancer Research. 2004; 10(14):4688-4698.

20. Carbone DP, Ciernik IF, Kelley MJ, Smith MC, Nadaf S, Kavanaugh D, Maher VE, Stipanov M, Contois D, Johnson BE, Pendleton CD, Seifert B, Carter C, Read EJ, Greenblatt J, Top LE, et al. Immunization with mutant p53- and K-ras-derived peptides in cancer patients: immune response and clinical outcome. Journal of clinical oncology : official journal of the American Society of Clinical Oncology. 2005; 23(22):5099-5107.

21. Hueman MT, Dehqanzada ZA, Novak TE, Gurney JM, Woll MM, Ryan GB, Storrer CE, Fisher C, McLeod DG, Ioannides CG, Ponniah S and Peoples GE. Phase I clinical trial of a HER-2/neu peptide (E75) vaccine for the prevention of prostate-specific antigen recurrence in high-risk prostate cancer patients. Clinical cancer research : an official journal of the American Association for Cancer Research. 2005; 11(20):7470-7479.

22. Gjertsen MK, Buanes T, Rosseland AR, Bakka A, Gladhaug I, Soreide O, Eriksen JA, Moller M, Baksaas I, Lothe RA, Saeterdal I and Gaudernack G. Intradermal ras peptide vaccination with granulocyte-macrophage colony-stimulating factor as adjuvant: Clinical and immunological responses in patients with pancreatic adenocarcinoma. International journal of cancer Journal international du cancer. 2001; 92(3):441-450.

23. Mizukoshi E, Nakagawa H, Kitahara M, Yamashita T, Arai K, Sunagozaka H, Fushimi K, Kobayashi E, Kishi H, Muraguchi A and Kaneko S. Immunological features of T cells induced by human telomerase reverse transcriptase-derived peptides in patients with hepatocellular carcinoma. Cancer letters. 2015; 364(2):98-105.

24. Rahma OE, Ashtar E, Czystowska M, Szajnik ME, Wieckowski E, Bernstein S, Herrin VE, Shams MA, Steinberg SM, Merino M, Gooding W, Visus C, Deleo AB, Wolf JK, Bell JG, Berzofsky JA, et al. A gynecologic oncology group phase II trial of two p53 peptide vaccine approaches: subcutaneous injection and intravenous pulsed dendritic cells in high recurrence risk ovarian cancer patients. Cancer immunology, immunotherapy : CII. 2012; 61(3):373-384.

25. Schuler PJ, Harasymczuk M, Visus C, Deleo A, Trivedi S, Lei Y, Argiris A, Gooding W, Butterfield LH, Whiteside TL and Ferris RL. Phase I dendritic cell p53 peptide vaccine for head and neck cancer. Clinical cancer research : an official journal of the American Association for Cancer Research. 2014; 20(9):2433-2444.

26. Wood LV, Fojo A, Roberson BD, Hughes MS, Dahut W, Gulley JL, Madan RA, Arlen PM, Sabatino M, Stroncek DF, Castiello L, Trepel JB, Lee MJ, Parnes HL, Steinberg SM, Terabe M, et al. TARP vaccination is associated with slowing in PSA velocity and decreasing tumor growth rates in patients with Stage D0 prostate cancer. Oncoimmunology. 2016; 5(8):e1197459.

27. Sabbatini P, Tsuji T, Ferran L, Ritter E, Sedrak C, Tuballes K, Jungbluth AA, Ritter G, Aghajanian C, Bell-McGuinn K, Hensley ML, Konner J, Tew W, Spriggs DR, Hoffman EW, Venhaus R, et al. Phase I trial of overlapping long peptides from a tumor self-antigen and poly-ICLC shows rapid induction of integrated immune response in ovarian cancer patients. Clinical cancer research : an official journal of the American Association for Cancer Research. 2012; 18(23):6497-6508.

28. Diefenbach CS, Gnjatic S, Sabbatini P, Aghajanian C, Hensley ML, Spriggs DR, Iasonos A, Lee H, Dupont B, Pezzulli S, Jungbluth AA, Old LJ and Dupont J. Safety and immunogenicity study of NY-ESO-1b peptide and montanide ISA-51 vaccination of patients with epithelial ovarian cancer in high-risk first remission. Clinical cancer research : an official journal of the American Association for Cancer Research. 2008; 14(9):2740-2748.

29. Kakimi K, Isobe M, Uenaka A, Wada H, Sato E, Doki Y, Nakajima J, Seto Y, Yamatsuji T, Naomoto Y, Shiraishi K, Takigawa N, Kiura K, Tsuji K, Iwatsuki K, Oka M, et al. A phase I study of vaccination with NY-ESO-1f peptide mixed with Picibanil OK-432 and Montanide ISA-51 in patients with cancers expressing the NY-ESO-1 antigen. International journal of cancer Journal international du cancer. 2011; 129(12):2836-2846.

30. Bender A, Karbach J, Neumann A, Jager D, Al-Batran SE, Atmaca A, Weidmann E, Biskamp M, Gnjatic S, Pan L, Hoffman E, Old LJ, Knuth A and Jager E. LUD 00-009: phase 1 study of intensive course immunization with NY-ESO-1 peptides in HLA-A2 positive patients with NY-ESO-1-expressing cancer. Cancer immunity. 2007; 7:16.

31. Barve M, Bender J, Senzer N, Cunningham C, Greco FA, McCune D, Steis R, Khong H, Richards D, Stephenson J, Ganesa P, Nemunaitis J, Ishioka G, Pappen B, Nemunaitis M, Morse M, et al. Induction of immune responses and clinical efficacy in a phase II trial of IDM-2101, a 10-epitope cytotoxic T-lymphocyte vaccine, in metastatic non-small-cell lung cancer. Journal of clinical oncology : official journal of the American Society of Clinical Oncology. 2008; 26(27):4418-4425.

32. Aruga A, Takeshita N, Kotera Y, Okuyama R, Matsushita N, Ohta T, Takeda K and Yamamoto M. Long-term Vaccination with Multiple Peptides Derived from Cancer-Testis Antigens Can Maintain a Specific T-cell Response and Achieve Disease Stability in Advanced Biliary Tract Cancer. Clinical cancer research : an official journal of the American Association for Cancer Research. 2013; 19(8):2224-2231.

33. Suzuki H, Fukuhara M, Yamaura T, Mutoh S, Okabe N, Yaginuma H, Hasegawa T, Yonechi A, Osugi J, Hoshino M, Kimura T, Higuchi M, Shio Y, Ise K, Takeda K and Gotoh M. Multiple therapeutic peptide vaccines consisting of combined novel cancer testis antigens and anti-angiogenic peptides for patients with non-small cell lung cancer. Journal of translational medicine. 2013; 11:97.

34. Kono K, Mizukami Y, Daigo Y, Takano A, Masuda K, Yoshida K, Tsunoda T, Kawaguchi Y, Nakamura Y and Fujii H. Vaccination with multiple peptides derived from novel cancer-testis antigens can induce specific T-cell responses and clinical responses in advanced esophageal cancer. Cancer science. 2009; 100(8):1502-1509.

35. Kono K, Iinuma H, Akutsu Y, Tanaka H, Hayashi N, Uchikado Y, Noguchi T, Fujii H, Okinaka K, Fukushima R, Matsubara H, Ohira M, Baba H, Natsugoe S, Kitano S, Takeda K, et al. Multicenter, phase II clinical trial of cancer vaccination for advanced esophageal cancer with three peptides derived from novel cancer-testis antigens. Journal of translational medicine. 2012; 10:141.

36. Welters MJ, Kenter GG, Piersma SJ, Vloon AP, Lowik MJ, Berends-van der Meer DM, Drijfhout JW, Valentijn AR, Wafelman AR, Oostendorp J, Fleuren GJ, Offringa R, Melief CJ and van der Burg SH. Induction of tumor-specific CD4+ and CD8+ T-cell immunity in cervical cancer patients by a human papillomavirus type 16 E6 and E7 long peptides vaccine. Clinical cancer research : an official journal of the American Association for Cancer Research. 2008; 14(1):178-187.

37. Rossmann E, Osterborg A, Lofvenberg E, Choudhury A, Forssmann U, von Heydebreck A, Schroder A and Mellstedt H. Mucin 1-specific active cancer immunotherapy with tecemotide (L-BLP25) in patients with multiple myeloma: an exploratory study. Human vaccines & immunotherapeutics. 2014; 10(11):3394-3408.

38. Sonpavde G, Wang M, Peterson LE, Wang HY, Joe T, Mims MP, Kadmon D, Ittmann MM, Wheeler TM, Gee AP, Wang RF and Hayes TG. HLA-restricted NY-ESO-1 peptide immunotherapy for metastatic castration resistant prostate cancer. Investigational new drugs. 2014; 32(2):235-242.

39. Pollack IF, Jakacki RI, Butterfield LH, Hamilton RL, Panigrahy A, Potter DM, Connelly AK, Dibridge SA, Whiteside TL and Okada H. Antigen-specific immune responses and clinical outcome after vaccination with glioma-associated antigen peptides and polyinosinic-polycytidylic acid stabilized by lysine and carboxymethylcellulose in children with newly diagnosed malignant brainstem and nonbrainstem gliomas. Journal of clinical oncology : official journal of the American Society of Clinical Oncology. 2014; 32(19):2050-2058.

40. Yoshitake Y, Fukuma D, Yuno A, Hirayama M, Nakayama H, Tanaka T, Nagata M, Takamune Y, Kawahara K, Nakagawa Y, Yoshida R, Hirosue A, Ogi H, Hiraki A, Jono H, Hamada A, et al. Phase II clinical trial of multiple peptide vaccination for advanced head and neck cancer patients revealed induction of immune responses and improved OS. Clinical cancer research : an official journal of the American Association for Cancer Research. 2015; 21(2):312-321.

41. Reyes D, Salazar L, Espinoza E, Pereda C, Castellon E, Valdevenito R, Huidobro C, Ines Becker M, Lladser A, Lopez MN and Salazar-Onfray F. Tumour cell lysate-loaded dendritic cell vaccine induces biochemical and memory immune response in castration-resistant prostate cancer patients. British journal of cancer. 2013; 109(6):1488-1497.

42. Tanaka T, Kitamura H, Inoue R, Nishida S, Takahashi-Takaya A, Kawami S, Torigoe T, Hirohashi Y, Tsukamoto T, Sato N and Masumori N. Potential survival benefit of anti-apoptosis protein: survivin-derived peptide vaccine with and without interferon alpha therapy for patients with advanced or recurrent urothelial cancer--results from phase I clinical trials. Clinical & developmental immunology. 2013; 2013:262967.

43. Hazama S, Nakamura Y, Takenouchi H, Suzuki N, Tsunedomi R, Inoue Y, Tokuhisa Y, Iizuka N, Yoshino S, Takeda K, Shinozaki H, Kamiya A, Furukawa H and Oka M. A phase I study of combination vaccine treatment of five therapeutic epitope-peptides for metastatic colorectal cancer; safety, immunological response, and clinical outcome. Journal of translational medicine. 2014; 12:63.

44. Kameshima H, Tsuruma T, Torigoe T, Takahashi A, Hirohashi Y, Tamura Y, Tsukahara T, Ichimiya S, Kanaseki T, Iwayama Y, Sato N and Hirata K. Immunogenic enhancement and clinical effect by type-I interferon of anti-apoptotic protein, survivin-derived peptide vaccine, in advanced colorectal cancer patients. Cancer science. 2011; 102(6):1181-1187.

45. Rahma OE, Hamilton JM, Wojtowicz M, Dakheel O, Bernstein S, Liewehr DJ, Steinberg SM and Khleif SN. The immunological and clinical effects of mutated ras peptide vaccine in combination with IL-2, GM-CSF, or both in patients with solid tumors. Journal of translational medicine. 2014; 12:55.

46. Kirner A, Mayer-Mokler A and Reinhardt C. IMA901: a multi-peptide cancer vaccine for treatment of renal cell cancer. Human vaccines & immunotherapeutics. 2014; 10(11):3179-3189.

47. Iversen TZ, Engell-Noerregaard L, Ellebaek E, Andersen R, Larsen SK, Bjoern J, Zeyher C, Gouttefangeas C, Thomsen BM, Holm B, Thor Straten P, Mellemgaard A, Andersen MH and Svane IM. Long-lasting disease stabilization in the absence of toxicity in metastatic lung cancer patients vaccinated with an epitope derived from indoleamine 2,3 dioxygenase. Clinical cancer research : an official journal of the American Association for Cancer Research. 2014; 20(1):221-232.

48. Okada H, Butterfield LH, Hamilton RL, Hoji A, Sakaki M, Ahn BJ, Kohanbash G, Drappatz J, Engh J, Amankulor N, Lively MO, Chan MD, Salazar AM, Shaw EG, Potter DM and Lieberman FS. Induction of robust type-I CD8+ T-cell responses in WHO grade 2 low-grade glioma patients receiving peptide-based vaccines in combination with poly-ICLC. Clinical cancer research : an official journal of the American Association for Cancer Research. 2015; 21(2):286-294.

49. Ott PA, Hu Z, Keskin DB, Shukla SA, Sun J, Bozym DJ, Zhang W, Luoma A, Giobbie-Hurder A, Peter L, Chen C, Olive O, Carter TA, Li S, Lieb DJ, Eisenhaure T, et al. An immunogenic personal neoantigen vaccine for patients with melanoma. Nature. 2017; 547(7662):217-221.

50. Borch TH, Engell-Noerregaard L, Zeeberg Iversen T, Ellebaek E, Met O, Hansen M, Andersen MH, Thor Straten P and Svane IM. mRNA-transfected dendritic cell vaccine in combination with metronomic cyclophosphamide as treatment for patients with advanced malignant melanoma. Oncoimmunology. 2016; 5(9):e1207842.

51. Sahin U, Derhovanessian E, Miller M, Kloke BP, Simon P, Lower M, Bukur V, Tadmor AD, Luxemburger U, Schrors B, Omokoko T, Vormehr M, Albrecht C, Paruzynski A, Kuhn AN, Buck J, et al. Personalized RNA mutanome vaccines mobilize poly-specific therapeutic immunity against cancer. Nature. 2017; 547(7662):222-226.

52. Bercovici N, Haicheur N, Massicard S, Vernel-Pauillac F, Adotevi O, Landais D, Gorin I, Robert C, Prince HM, Grob JJ, Leccia MT, Lesimple T, Wijdenes J, Bartholeyns J, Fridman WH, Salcedo M, et al. Analysis and characterization of antitumor T-cell response after administration of dendritic cells loaded with allogeneic tumor lysate to metastatic melanoma patients. Journal of immunotherapy. 2008; 31(1):101-112.

53. Ellebaek E, Engell-Noerregaard L, Iversen TZ, Froesig TM, Munir S, Hadrup SR, Andersen MH and Svane IM. Metastatic melanoma patients treated with dendritic cell vaccination, Interleukin-2 and metronomic cyclophosphamide: results from a phase II trial. Cancer immunology, immunotherapy : CII. 2012; 61(10):1791-1804.

54. Zeestraten EC, Speetjens FM, Welters MJ, Saadatmand S, Stynenbosch LF, Jongen R, Kapiteijn E, Gelderblom H, Nijman HW, Valentijn AR, Oostendorp J, Fathers LM, Drijfhout JW, van de Velde CJ, Kuppen PJ, van der Burg SH, et al. Addition of interferon-alpha to the p53-SLP(R) vaccine results in increased production of interferon-gamma in vaccinated colorectal cancer patients: a phase I/II clinical trial. International journal of cancer Journal international du cancer. 2013; 132(7):1581-1591.

55. Kaufman HL, Lenz HJ, Marshall J, Singh D, Garett C, Cripps C, Moore M, von Mehren M, Dalfen R, Heim WJ, Conry RM, Urba WJ, Benson AB, 3rd, Yu M, Caterini J, Kim-Schulze S, et al. Combination chemotherapy and ALVAC-CEA/B7.1 vaccine in patients with metastatic colorectal cancer. Clinical cancer research : an official journal of the American Association for Cancer Research. 2008; 14(15):4843-4849.

56. McNeel DG, Becker JT, Eickhoff JC, Johnson LE, Bradley E, Pohlkamp I, Staab MJ, Liu G, Wilding G and Olson BM. Real-time immune monitoring to guide plasmid DNA vaccination schedule targeting prostatic acid phosphatase in patients with castration-resistant prostate cancer. Clinical cancer research : an official journal of the American Association for Cancer Research. 2014; 20(14):3692-3704.

57. Alfaro C, Perez-Gracia JL, Suarez N, Rodriguez J, Fernandez de Sanmamed M, Sangro B, Martin-Algarra S, Calvo A, Redrado M, Agliano A, Gonzalez A, Rodriguez I, Bolanos E, Hervas-Stubbs S, Perez-Calvo J, Benito A, et al. Pilot clinical trial of type 1 dendritic cells loaded with autologous tumor lysates combined with GM-CSF, pegylated IFN, and cyclophosphamide for metastatic cancer patients. Journal of immunology. 2011; 187(11):6130-6142.

58. McNeel DG, Chen YH, Gulley JL, Dwyer AJ, Madan RA, Carducci MA and DiPaola RS. Randomized phase II trial of docetaxel with or without PSA-TRICOM vaccine in patients with castrate-resistant metastatic prostate cancer: A trial of the ECOG-ACRIN cancer research group (E1809). Human vaccines & immunotherapeutics. 2015; 11(10):2469-2474.

59. Suzuki N, Hazama S, Ueno T, Matsui H, Shindo Y, Iida M, Yoshimura K, Yoshino S, Takeda K and Oka M. A phase I clinical trial of vaccination with KIF20A-derived peptide in combination with gemcitabine for patients with advanced pancreatic cancer. Journal of immunotherapy. 2014; 37(1):36-42.

60. Carmichael MG, Benavides LC, Holmes JP, Gates JD, Mittendorf EA, Ponniah S and Peoples GE. Results of the first phase 1 clinical trial of the HER-2/neu peptide (GP2) vaccine in disease-free breast cancer patients: United States Military Cancer Institute Clinical Trials Group Study I-04. Cancer. 2010; 116(2):292-301.

61. Babatz J, Rollig C, Lobel B, Folprecht G, Haack M, Gunther H, Kohne CH, Ehninger G, Schmitz M and Bornhauser M. Induction of cellular immune responses against carcinoembryonic antigen in patients with metastatic tumors after vaccination with altered peptide ligand-loaded dendritic cells. Cancer immunology, immunotherapy : CII. 2006; 55(3):268-276.

62. Mehrotra S, Britten CD, Chin S, Garrett-Mayer E, Cloud CA, Li M, Scurti G, Salem ML, Nelson MH, Thomas MB, Paulos CM, Salazar AM, Nishimura MI, Rubinstein MP, Li Z and Cole DJ. Vaccination with poly(IC:LC) and peptide-pulsed autologous dendritic cells in patients with pancreatic cancer. Journal of hematology & oncology. 2017; 10(1):82.
