# Supplementary figures and images for: Clinical activity of a htert (vx-001) cancer vaccine as post-chemotherapy maintenance immunotherapy in patients with stage IV non-small cell lung cancer: final results of a randomised phase 2 clinical trial
Source: Br J Cancer. 2020 Mar 25;122(10):1461–6. doi: 10.1038/s41416-020-0785-y (PMC7217860; doi:10.1038/s41416-020-0785-y)

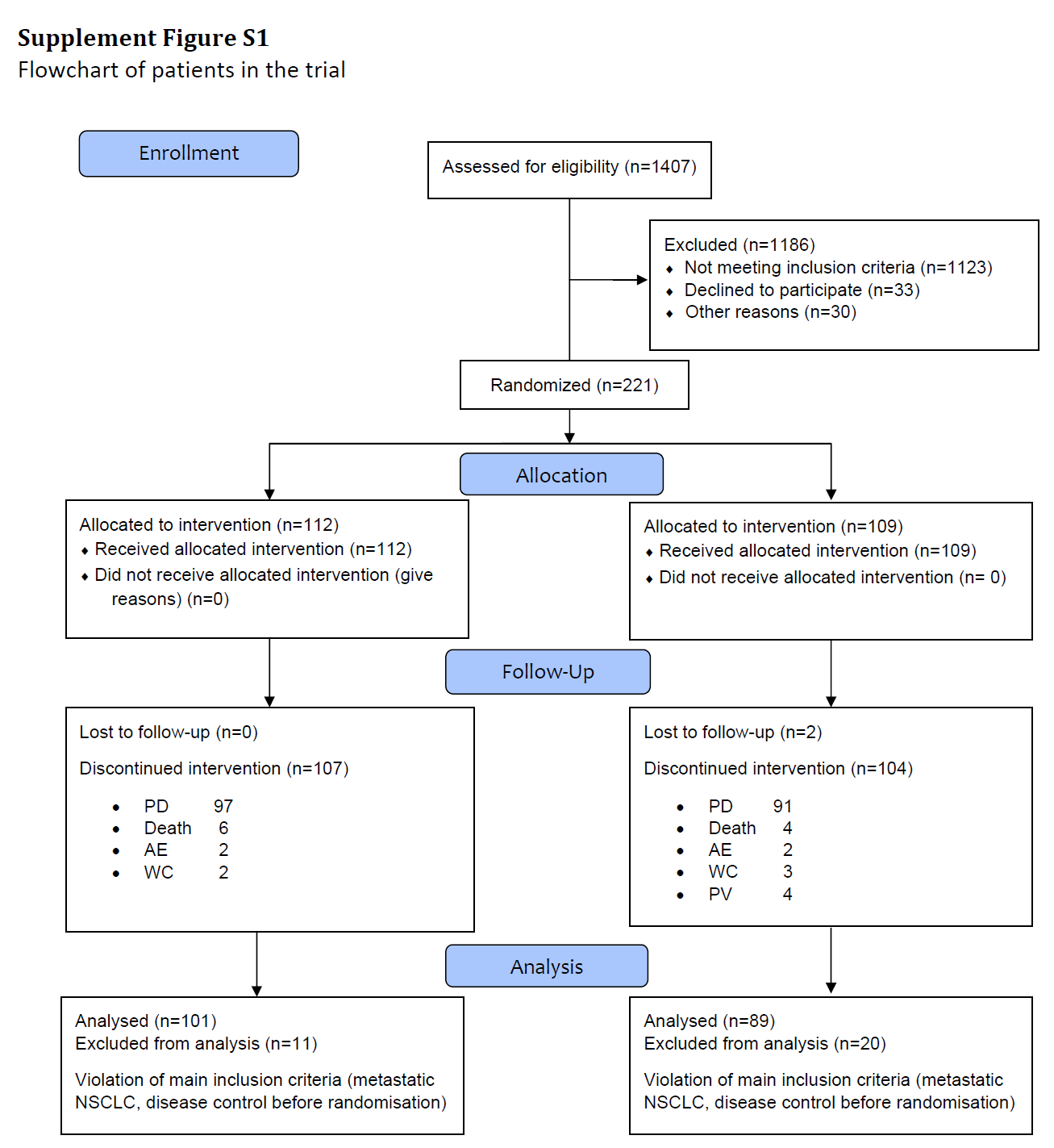

Supplement: Supplementary file 2 — Suppl.Fig.S1 [file 41416_2020_785_MOESM2_ESM.tif]
