# Supplementary material for: Clinical activity of a htert (vx-001) cancer vaccine as post-chemotherapy maintenance immunotherapy in patients with stage IV non-small cell lung cancer: final results of a randomised phase 2 clinical trial
Source: Br J Cancer. 2020 Mar 25;122(10):1461–6. doi: 10.1038/s41416-020-0785-y (PMC7217860; doi:10.1038/s41416-020-0785-y)
Supplement: Supplementary file 3 — Statistical Analysis Plan [file 41416_2020_785_MOESM3_ESM.docx]

| Statistical Analysis Plan |
| --- |
| A multicenter, randomized, double-blind, placebo-controlled Phase IIb Efficacy Study of Vx-001, a peptide-based cancer vaccine aimed to maintain disease control after first line treatment in HLA-A*0201 positive patients with TERT positive NSCLC (stage IV or recurrent stage I-III)  Vx-001-201 |
|  |

**Versions History**

| **Version Number** | **Date** | **Detail** |
| --- | --- | --- |
| Final Version 3.0 | 13 September 2016 | Current version: Update of the SAP in order to add Figures and complete exploratory analysis |
| Final Version 2.0 | 16 March 2016 | Current version: Update of the SAP following protocol amendment and modification of planned primary analysis |
| Final version 01 | 10 February 2013 | First final version of the SAP  authored by Ladislav Pecen, PhD, Study Statistician, SIRO Clinpharm |
|  |  |  |

Signatures

**Author**

**Venn Life Sciences**

|  |  |  |  |
| --- | --- | --- | --- |
| Name | Nelly Braquet/Antoinette Anger |  | Date |
| Position | Biostatistician |  |  |
| Company | Venn Life Sciences |  |  |

**Approval**

**Venn Life Sciences**

|  |  |  |  |
| --- | --- | --- | --- |
| Name | François Aubin |  | Date |
| Position | Head of Biometry, Medical and Methodology Expert |  |  |
| Company | Venn Life Sciences |  |  |

**Sponsor**

|  |  |  |  |
| --- | --- | --- | --- |
| Name | Kostas Kosmatopoulos MD, PhD |  | Date |
| Position | Chief Executive Officer |  |  |
| Company | Vaxon Biotech |  |  |

Contents

Abbreviations and definitions 6

1 Introduction 8

2 Highlights from study protocol 8

2.1 Background/Rationale 8

2.2 Study Objectives 8

2.2.1 Primary objective(s) 8

2.2.2 Secondary objectives 9

2.2.3 Exploratory objectives 9

2.2.4 Safety objectives 9

2.3 Investigational plan 10

2.3.1 Study design and randomisation 10

2.3.1 Blinding and breaking the study blind 13

2.3.2 Determination of sample size 13

2.3.2.1 Initial sample-size calculation: 13

2.3.2.2 Revised sample-size calculation: 14

2.3.3 Study assessments and study plan 15

3 Analysis datasets 18

3.1 Reasons for excluding patients from analysis datasets 18

3.1.1 Major protocol deviations 18

3.1.2 Minor protocol deviations 18

3.1.3 Study treatment discontinuations - Study discontinuations 19

3.2 Primary efficacy dataset: Full Analysis Set (FAS) 19

3.3 Per-protocol (PP) dataset 20

3.4 Safety (SAF) dataset 21

4 Endpoints for analysis 21

4.1 Efficacy endpoints 21

4.1.1 Primary efficacy endpoint(s) 21

4.1.2 Secondary efficacy endpoints 21

4.2 Safety endpoints 21

4.2.1 Adverse events 21

4.2.2 Laboratory endpoints 22

4.2.3 Other safety endpoints 23

4.2.3.1 Physical examination and Vital signs 23

4.2.3.2 ECG 23

4.2.3.3 ECOG 23

4.2.3.4 Concomitant medication 24

5 Statistical and Analytical Methods 24

5.1 General considerations 24

5.1.1 Presentation of results 24

5.1.2 Significance testing and estimation 24

5.2 Planned analysis 25

5.2.1 Demographics and baseline characteristics 25

5.2.2 Patient disposition and study discontinuations 26

5.2.3 Extent of exposure and compliance 26

5.2.4 Efficacy analyses 27

5.2.4.1 Primary efficacy analysis 27

5.2.4.2 Sensitivity efficacy analysis 27

5.2.4.3 Secondary efficacy analyses 28

5.2.4.4 Exploratory efficacy analyses 29

5.2.4.5 Other Exploratory Analyses 30

5.2.5 Safety analyses 30

5.2.5.1 Adverse events 30

5.2.5.2 Laboratory safety variables 32

5.2.5.1 Vital signs 33

5.2.5.2 Physical examinations 33

5.2.5.1 Injection site inspection 33

5.2.5.1 ECG 34

5.2.5.1 ECOG performance status 34

5.2.5.2 Prior and Concomitant medication 34

5.3 Statistical/Analytical issues 34

5.3.1 Adjustments for Covariates 34

5.3.2 Handling of Dropouts or Missing Data 35

5.3.3 Interim Analyses and Data Monitoring 36

5.3.4 Multicentre studies 36

5.3.5 Multiple Comparison/Multiplicity 36

5.3.6 Use of an "Efficacy Subset" of Patients 37

5.3.7 Active-Control Studies Intended to Show Equivalence 37

5.3.8 Examination of Subgroups 37

5.4 Data handling conventions 38

5.4.1 Baseline definitions 38

5.4.2 Retest, Outliers 38

5.4.2.1 Retests 38

5.4.2.2 Outliers 39

5.4.3 Windows for time points 39

5.4.4 Unscheduled visits 40

6 Modifications from the statistical sections in the protocol 40

7 Software documentation 42

8 Derived data 42

9 References 43

10 Appendices 44

10.1 Appendix 1: Statistical Tables List 44

10.2 Appendix 2: Statistical Figures List 46

10.3 Appendix 3: Individual Data Listings List 46

10.4 Appendix 4: TNM Clinical Classification of Lung Cancer 48

10.5 Appendix 5: ECOG Performance Status 50

10.6 Appendix 6: Revised RECIST guideline (version 1.1) 51

10.7 Appendix 7: U.S. National Cancer Institute (NCI) Common Terminology Criteria for Adverse Events (CTCAE) 56

Abbreviations and definitions

| **Ad** | Adenocarcinoma |
| --- | --- |
| **AE(s)** | Adverse Event(s) |
| **ALP** | ALkaline Phosphatase |
| **ALT (SGPT)** | ALanine aminoTransferase |
| **APC** | Antigen Presenting Cell |
| **ARG** | Arginine |
| **AST (SGOT)** | ASpartate aminoTransferase |
| **BUN** | Blood Urea Nitrogen |
| **CD** | Cluster of Differentiation |
| **CR** | Complete Response |
| **CRA** | Clinical Research Associate |
| **CT** | Computed Tomography |
| **CTL** | Cytotoxic T Lymphocyte |
| **DC** | Disease Control |
| **dL** | Deciliter (100ml) |
| **DNA** | DeoxyriboNucleic Acid |
| **DSR** | Data Summary Report |
| **DSMB** | Data Safety and Monitoring Board |
| **ECG** | ElectroCardioGram |
| **ECOG** | Eastern Cooperative Oncology Group |
| **eCRF** | electronic Case Report Form |
| **ELISPOT** | Enzyme-LInked immuno-Sorbent spot assay |
| **FAS** | Primary Analysis Set |
| **FDA** | Food and Drug Administration |
| **GCP** | Good Clinical Practice |
| **GCSP** | Global Clinical Safety and Pharmacovigilance |
| **GMP** | Good Manufacturing Practice |
| **GM-CSF** | Granulocyte-Macrophage Colony Stimulating Factor |
| **HLA** | Human Leukocyte Antigen |
| **HLA I** | Human Leukocyte Antigen class I |
| **HPLC** | High Pressure Liquid Chromatography |
| **IB** | Investigator Brochure |
| **ICF** | Informed Consent Form |
| **ID** | IDentification |
| **IEC** | Independent Ethics Committee |
| **IFA** | Incomplete Freund Adjuvant |
| **IFN-γ** | Interferon-gamma |
| **IL** | InterLeukin (subtypes IL-2, IL-4 etc) |
| **IND** | Investigational New Drug |
| **IRB** | Institutional Review Board |
| **ISR** | Injection Site Reaction |
| **ITT** | Intent-To-Treat |
| **IV** | IntraVenous |
| **IWRS** | Interactive Web Response System |
| **LCC** | Large Cell Carcinoma |
| **LD** | Longest Diameter |
| **LLT** | Lower Level Term |
| **mAb** | monoclonal Antibody |
| **MedDRA** | Medical Dictionary for Regulatory Activities |
| **MHC** | Major Histocompatibility Complex |
| **MRI** | Magnetic Resonance Imaging |
| **MTD** | Maximum Tolerated Dose |
| **NCI-CTCAE** | National Cancer Institute - Common Terminology Criteria for Adverse Events |
| **NE** | Not Evaluable |
| **NSCLC** | Non-Small Cell Lung Cancer |
| **OR** | Overall Response |
| **OS** | Overall Survival |
| **PBMC** | Peripheral Blood Mononuclear Cell |
| **PD** | Progressive Disease |
| **PET** | Positron Emission Tomography |
| **PFS** | Progression-Free Survival |
| **PP** | Per Protocol |
| **PR** | Partial Response |
| **PT** | Preferred Term |
| **PV** | PharmacoVigilance |
| **QC** | Quality Control |
| **RA** | Relative Affinity |
| **RECIST** | Response Evaluation Criteria In Solid Tumors |
| **RT** | RadioTherapy |
| **SAE** | Serious Adverse Event |
| **SAP** | Statistical Analysis Plan |
| **SCC** | Squamous Cell Carcinoma |
| **SCLC** | Small Cell Lung Cancer |
| **SD** | Stable Disease |
| **SM** | Study Manual |
| **SOC** | System Organ Class |
| **SSR** | Sample Size Reestimation |
| **TCR** | T-Cell Receptor |
| **TEAE** | Treatment-Emergent Adverse Event |
| **TERT** | TElomerase Reverse Transcriptase |
| **TESAE** | Treatment-Emergent Serious Adverse Event |
| **TYR** | Tyrosine |
| **ULN** | Upper Limit of Normal |
| **vs** | versus |
| **WBC** | White Blood Cells |
| **WHO** | World Health Organization |

# Introduction

This document is the statistical analysis plan (SAP) for the VaxLung Vx-001-201 study. The purpose of this SAP is to provide a comprehensive and detailed description of the statistical analyses that will be carried out to assess the clinical efficacy and safety of the study treatment, as outlined in the latest version of the study protocol, dated 07 July 2015. The SAP pre-specifies the statistical approaches to be used and is validated prior to the study database lock and the unblinding of the randomisation schedule to ensure the credibility of the study findings.

This is the third version of the SAP with added figures and completed exploratory analysis. The second version of the SAP, developed following a protocol amendment aiming at modifying the planned primary analysis was finalised on 16^th^ March 2016. The first version of the SAP was finalised on 10^th^ February 2015.

# Highlights from study protocol

## Background/Rationale

Full details of the background and rationale for the study are provided in Section 1 of the protocol.

## Study Objectives

### Primary objective(s)

The efficacy objective of the study is to demonstrate the clinical efficacy of Vx-001 vs placebo, as maintenance treatment after first-line treatment in NSCLC patients with disease control (SD, PR or CR).

The primary objective is a time-to-event comparison of overall survival in Vx-001 treated vs placebo treated patients.

### Secondary objectives

The secondary objectives are:

- Comparison of survival rate at 12 months in Vx-001 treated vs placebo treated patients,
- Comparison of Time to Treatment Failure in Vx-001 treated vs placebo treated patients

### Exploratory objectives

The exploratory objectives are:

- Comparison of disease control rate after the end of subsequent second line treatments between Vx-001 treated vs placebo treated patients,
- Comparison of vaccine induced immune responses between Vx-001 vs placebo in terms of frequency of TERT_572_ specific IFN-γ and Perforin producing T cells in the blood of patients. Immune response will be evaluated before treatment, after the second and sixth injections or at end of treatment in case of patient withdrawal from the study before or at Week 18 and from week 39 after every two injections.
- Comparison of overall survival in patients with vaccine induced immune response detected after the second and sixth injection of Vx-001 vs:
  - patients who received at least two and six injections of placebo, respectively,
  - patients randomised to Vx-001 who did not develop vaccine specific immune response after the second and sixth injections of Vx-001, respectively.
- Comparison of overall survival in Vx-001-treated vs placebo-treated patients who had:
  - a vaccine specific immune response before Vx-001 or placebo injection,
  - high level of TERT expression or low levels of TERT expression in their tumour biopsy.

### Safety objectives

The safety objective is to compare the safety and tolerability of Vx-001 treated *vs* placebo treated patients.

The following parameters of safety and tolerability are assessed:

- Physical examination and vital signs
- Electrocardiogram
- Adverse event evaluation
- ECOG performance status (see Appendix 5: 10.5)
- Clinical laboratory evaluation: hematology and clinical chemistry

## Investigational plan

### Study design and randomisation

This international, multicentre (approximately 76 sites in European Union countries), phase II study is conducted according to a double-blind, placebo-controlled, randomized (1:1 ratio, i.e. 1 Vx-001 and adjuvant vs 1 placebo and adjuvant) design.

The aim of the study is to assess the efficacy of Vx-001 vaccination as maintenance treatment in patients after 4 cycles of first-line chemotherapy treatment. Patients should have documented stage IV NSCLC as defined by IASLC Lung Cancer Staging Project (7th edition) or recurrent stage I-III disease at least 6 months after initial treatment, or after the end of adjuvant chemotherapy or after standard locoregional treatment as defined by the American College of Chest Physicians. To be eligible, patients entering the study should have documented either stable disease or objective response (PR or CR) after first line chemotherapy, will have to be HLA-A*0201 positive and their tumor will have to be TERT-positive.

Patients are administered at predefined visits the Vx-001 + Montanide ISA51 adjuvant subcutaneously, at a dose of 2mg, or placebo + Montanide ISA51 adjuvant subcutaneously. The vaccination protocol comprises of two injections with the TYR-Vx001 or placebo, one at day 0 and another at week 3, and four injections with the ARG-Vx001 or placebo, at weeks 6, 9, 12 and 15.

Following the treatment assessment at week 18, patients receive the ARG-Vx001 or placebo, every 12 weeks starting from week 27 until treatment discontinuation.

The patient may discontinue treatment for one of the following reasons:

- Progression assessed by CT scan and according to RECIST criteria,
- Adverse event, including clinical adverse event, unexpected toxicity, clinically significant biological or physical examination abnormality,
- Death, to be considered as an adverse event within 28 days following the vaccination,
- Major protocol violation,
- Withdrawal of consent,
- Other reason.

All patients withdrawn from the study for any reason must undergo a complete final study termination visit and enter in the follow-up period. If the patient discontinues the study due to a study drug-related adverse event, s/he must be followed

The maximum duration of the treatment period for any patient for this study (comprising of the entire period of vaccinations until progression or withdrawal for any other reason) will be no more than the duration of the study. The duration of active follow up for survival and disease recurrence related to study participation will be dictated by the completion of the final analysis. Upon completion of the final analysis and if appropriate, patients under active treatment will continue to receive Vx-001 (the ARG-Vx001 peptide) within the remit of the study, until disease progression or marketing authorization of the vaccine and at the latest until the end of 2018. During this period patients will be followed-up every 12 weeks as described in the protocol. Patients under placebo treatment will stop to receive placebo and must undergo a complete final study termination visit and enter in the follow-up period for collection of survival data.

Treatment administration and duration:

- Investigational product:

Patients receive two vaccinations with 2mg TYR-Vx001 (weeks 0, 3), four vaccinations with 2mg ARG-Vx001 (weeks 6, 9, 12, 15) followed by vaccinations with 2mg ARG-Vx001 every 12 weeks commencing at week 27 until disease progression. If appropriate, patients, that have not progressed, will continue to receive Vx-001, until disease progression, unacceptable adverse events, death, major protocol violation, withdrawal of consent, or other reason as study closure.

Solutions for injection of Vx-001 peptides will be emulsified with adjuvant Montanide ISA51™ just prior to injection and will be administered subcutaneously.

- Reference therapy: Placebo:

Patients receive two subcutaneous injections with TYR-Vx001 Placebo at weeks 0, 3, followed by four vaccinations with ARG-Vx001 Placebo (weeks 6, 9, 12, 15) followed by injections of ARG-Vx001 Placebo at 12-week intervals commencing at week 27 until disease progression, unacceptable adverse events, death, major protocol violation, withdrawal of consent, or other reason as study closure, or study unblinding, if earlier.

Placebo solutions for injection will be emulsified with Montanide ISA51™ and will be administered subcutaneously.

The vaccination schedule is detailed in Figure 1.

Figure 1: Detailed schedule of vaccinations

Randomization:

Patients are randomized (stratified block randomization) in the ratio 1:1 to either the investigational arm (Vx-001) or the placebo arm, following the completion of all screening assessments and after confirmation of their eligibility. The randomization is stratified on:

- Response to first line chemotherapy (stable disease vs objective response)
- Squamous vs non squamous
- Stage IV vs recurrent stage I-III NSCLC

The purpose of stratification is to ensure even distribution of patients between the two study arms, with respect to the strata. Randomization is performed via an interactive web response system (IWRS).

### Blinding and breaking the study blind

The study is double-blind (i.e. both investigators and patients are blind with respect to the treatment used). In addition, the randomization code is kept blind to the Sponsor, the individuals monitoring the study and the data-management and biostatistics teams.

Emergency unblinding should only occur in the case of medical events where the investigator or the physician in charge of the patient feels that the clinical management of the patient would be significantly compromised by not knowing the identity of the allocated study drug. The investigator or designee, should contact Vaxon Biotech’s Medical Monitor either directly or through the local safety contact to discuss the need for emergency unblinding. Once agreed, code-breaking is to be carried out via the IWRS. The reasons for unblinding, date and time must be documented by the investigator. Notification of the Sponsor occurs automatically via the IWRS without giving the result of the unbinding. The Pharmacovigilance Group of the Sponsor or its designee also have procedures in place for emergency unblinding, if needed.

If the code has been broken and there are no medical reasons for discontinuation, the patient may continue the study treatment. In case of withdrawal from study treatment the patient is to be followed up for survival.

Final unblinding and code breaking will occur after the database lock for the primary analysis, planned when the last randomized patient will have reached 6 month follow-up post first vaccination (see sections 5.2.4.1 and 6).

### Determination of sample size

#### **Initial sample-size calculation:**

The initial sample-size calculation, at the time the first version of the protocol was finalized was the following:

The number of patients to enrol is estimated based on the following hypotheses:

H0: The proportion of patients with OS after 12 months of treatment in the Vx-001 group is less than or equal to that of the placebo group.

H1: The proportion of patients with OS after 12 months of treatment in the Vx-001 group is greater than that of the placebo group.

The proportion of patients in the placebo group with OS at 12 months from randomization is assumed to be **43%** (Fidias et al 2009; Ciuleanu et al 2009; Cappuzzo et al., 2010). The proportion of patients in the vaccine group with OS at 12 months from the end of first-line treatment is assumed to be **58%**.

The primary analysis will be performed after all patients have been followed up for at least 12 months. Based on the above assumptions for OS at 12 months and 200 evaluable patients (100 on the Vx-001 arm and 100 on the placebo arm), the primary efficacy analysis would have an overall power of approximately 80% to detect a difference in treatment effect at the 0.1 one-sided significance level.

Although the full analysis set (FAS, see definition in the statistical methods section) is the primary efficacy dataset, the study will be powered based on the per protocol (PP) analysis of the primary efficacy endpoint. Assuming that all randomised patients will be in the FAS, and assuming that 10% of patients in the FAS will be excluded from the PP dataset, a total of 220 patients will be randomised in the study to attain approximately 200 evaluable patients.

The study design is based on comparison of proportions in two treatment arms using an one-sided a Pearson’s chi-squared test.

#### **Revised sample-size calculation:**

The sample size-calculation was revised as follows, following the change in the primary efficacy analysis

Assuming 50% of screened patients will experience disease control after 1st line chemotherapy, 41% of screened patients will be HLA-A*0201 positive, of whom 80% will be TERT positive in their tumour biopsy, approximately 1500 patients will be screened to enrol around 220 HLA-A*0201 positive patients with TERT expressing NSCLC with disease control after 1st line treatment (110 on the Vx-001 arm and 110 on the placebo arm), calculated using SAS® 9.2 software with a maximum of 230 patients.

The number of patients to enroll is estimated based on the following hypotheses:

H0: The proportion of patients with OS after 12 months of treatment in the Vx-001 group is less than or equal to that of the placebo group.

H1: The proportion of patients with OS after 12 months of treatment in the Vx-001 group is greater than that of the placebo group.

The proportion of patients in the placebo group with OS at 12 months from randomization is assumed to be 43% (Fidias et al 2009; Ciuleanu et al 2009; Cappuzzo et al., 2010), corresponding to an overall survival of 9.8 months, assuming exponential survival. The proportion of patients in the vaccine group with OS at 12 months from the end of first-line treatment is assumed to be 58% corresponding to an overall survival of 15.2 months, assuming exponential survival.

The primary analysis will consist in a comparison of overall survival between the two arms using the Kaplan Meier method to build survival curves and the Logrank test to compare them between treatment groups and will be performed after all patients have been followed up for 6 months. The follow-up of the patients will nevertheless continue after this primary analysis until 12 months, as planned. Based on the above assumptions for OS at 12 months and 200 evaluable patients (100 on the Vx-001 arm and 100 on the placebo arm), the primary efficacy analysis will have an overall power of approximately 82.5% to detect a difference in treatment effect at the 0.05 one sided significance level.

Although the full analysis set (FAS, see definition in Section 3) is the primary efficacy dataset, the study will be powered based on the per protocol (PP) analysis of the primary efficacy endpoint.

Assuming that all randomised patients will be in the FAS, and assuming that 10% of patients in the FAS will be excluded from the PP dataset, a total of 220 patients will be randomised in the study to attain approximately 200 evaluable patients.

The study design is based on comparison of survival curves between the two treatment arms using a one-sided Logrank test.

The recruitment time is estimated to be 39 months (5.6 patients per month on average), the follow-up is 12 months, making the total duration of the study approximately 51 months.

### Study assessments and study plan

One written Informed Consent Form (ICF) with two agreements (one to take part to the screening procedures and one to take part to the study) must be obtained. The first agreement documents the agreement to participate to the screening procedures, it should be obtained prior to initiating any screening specific assessments. The second agreement can be obtained at the screening procedure and at the latest at baseline visit and before the randomization. This second agreement documents the agreement to participate to the study. Only patients HLA-A*0201 positive with TERT expressing tumor and who have documented disease control can be randomized.

All baseline evaluations must be completed prior to initiating treatment with study medication.

Medical and physical examinations should be performed by a qualified physician and should include a thorough review of all body systems. Relevant data will be captured in the electronic Case Report Form (eCRF). All data will be recorded in source documents.

The study consists of the following phases/visits:

- Screening evaluation phase (HLA-A*0201 typing is necessary prior to any further screening, followed by assessment of TERT positivity of the tumor - access to tumor tissue biopsy is a pre-requisite. HLA-A2 patients with TERT expressing tumors will perform a CT scan / MRI to document disease control)
- Baseline visit, randomization and treatment allocation (Day 1 of week 0)
- Treatment period (visits every 3 weeks from randomization to week 15 and every 12 weeks from week 27 until the end of study treatment)
- Treatment assessment at week 18
- End of treatment (final) visit
- Survival follow-up contacts after the end of vaccination treatment (every 12 weeks, can be done by phone).

Patients have to visit the clinic/institution for investigational product administration and assessments according to the Flow Chart and Schedule of Assessments (see below).

|  | **Procedures** | **Screening period *** | **Baseline***** | **Treatment Period**  **Vaccination every 3 weeks** | | | | | **Treatment Evaluation** | **Treatment period**  **Vaccination every 12 weeks** | | **End of treatment (EOT)** | **Post Treatment Follow-Up Every 12 weeks** |
| --- | --- | --- | --- | --- | --- | --- | --- | --- | --- | --- | --- | --- | --- |
| **Time (weeks)** |  | Up to 3 weeks after last chemotherapy administration | 0 | 3 | 6 | 9 | 12 | 15 | 18 weeks | 27, 52, 75, and every 24 weeks until EOT | 39, 63 and every 24 weeks until EOT | Final visit | Survival follow-up |
| **Vaccination** |  |  | **_TYR-Vx001_** | **_TYR-Vx001_** | **_ARG-Vx001_** | **_ARG-Vx001_** | **_ARG-Vx001_** | **_ARG-Vx001_** |  | **_ARG-Vx001_** | **_ARG-Vx001_** |  |  |
| **Eligibility** | Informed consent | X | X^1^ |  |  |  |  |  |  |  |  |  |  |
|  | Inclusion/exclusion criteria | X | X |  |  |  |  |  |  |  |  |  |  |
|  | Blood sample for HLA typing | X |  |  |  |  |  |  |  |  |  |  |  |
|  | Tumour sample for TERT and biomarker | X |  |  |  |  |  |  |  |  |  |  |  |
|  | Allocation of IWRS subject number | X |  |  |  |  |  |  |  |  |  |  |  |
| **Randomization** | Randomization |  | X |  |  |  |  |  |  |  |  |  |  |
| **Study Drug** | **Vx-001 - Placebo** |  | X | X | X | X | X | X |  | X | X |  |  |
| **Safety** | Urinary pregnancy test |  | X |  |  |  |  |  |  |  |  | X^2^ |  |
|  | Demography, medical and treatment history |  | X |  |  |  |  |  |  |  |  |  |  |
|  | Physical exam, vital signs |  | X | X | X | X | X | X | X | X | X | X |  |
|  | ECOG Performance Status |  | X |  |  | X |  |  | X | X |  | X |  |
|  | Injection-site inspection |  | X | X | X | X | X | X | X | X | X | X |  |
|  | Body weight  (height at baseline) |  | X |  |  |  |  |  | X | X | X | X |  |
|  | 12-Lead ECG |  | X |  |  |  |  |  | X |  |  | X |  |
|  | Adverse Events/Toxicity assessments | X**** | X | X | X | X | X | X | X | X | X | X |  |
|  | Record concomitant medications |  | X | X | X | X | X | X | X | X | X | X |  |
|  | Haematology^3^ & clinical chemistry^4^ |  | X^6^ |  |  | X |  |  | X | X | X | X |  |
| **Efficacy** | Chest /abdomen CT or MRI | X** |  |  |  |  |  |  |  |  |  |  |  |
|  | Bone scanning^5^ | X |  |  |  |  |  |  |  |  |  |  |  |
|  | Blood sample (immunomonitoring) and biomarkers)^7^ |  | X |  | X |  |  |  | X |  | X | X^8^ |  |
| **Survival** | Record post-study treatments and responses |  |  |  |  |  |  |  |  |  |  |  | X |

.

# Analysis datasets

The manner in which specific irregularities arising during the study will be dealt with to define the FAS and PP analysis set (including the classification of deviations/violations as “major” and levels of treatment compliance as “good”) will be clearly justified and documented in the data-review meeting minutes prior to treatment unblinding and presented in the clinical study report along with a discussion of any potential sources of bias.

## Reasons for excluding patients from analysis datasets

Protocol deviations will be identified and their list will be finalised prior to the data review. The list of deviations will be prepared by Venn Life Sciences and approved by the sponsor. The sponsor will be responsible for classifying all deviations as either major or minor.

### **Major protocol deviations**

Major protocol deviations are defined as deviations liable to prevent or change the interpretation of the results of the primary efficacy analysis of the study. The following deviations may be considered as major (this list is not exhaustive and will be reviewed at the time of the blind review meeting):

- non compliance with the inclusion or non inclusion criteria
- non compliance with the randomisation procedure
- non compliance with study treatment
- no post-baseline data for the primary efficacy endpoint
- intake of forbidden medication

### **Minor protocol deviations**

All other deviations will, *a priori*, be considered as minor deviations. However, all deviations will be reviewed and adjudicated as either major or minor during the blind review meeting before database lock and code break.

### **Study treatment discontinuations - Study discontinuations**

Patients may voluntarily discontinue participation in this study at any time without giving reason(s). The investigator may also, at his/her discretion, withdraw the patient from the study at any time.

The patient may discontinue treatment for one of the following reason:

- Progression assessed by CT scan and according to RECIST criteria,
- Adverse event, including clinical adverse event, unexpected toxicity, clinically significant biological or physical exam abnormality,
- Death, to be considered as an adverse event if it occurred within 4 weeks after a vaccination,
- Major protocol violation,
- Withdrawal of consent,
- Other reason.

All patients withdrawn from the study for any reason must undergo a complete final study termination visit and enter in the follow-up period.

If the patient discontinues the study due to a study drug-related adverse event, s/he must be followed weekly until resolution or stabilization of the event, whichever occurs first.

Once the patient has discontinued the investigational treatment, the date of discontinuation and reason(s) must be documented in the patient’s medical records and eCRF. Assessments for the final visit, should be performed. Once a patient discontinues the randomized therapy, the patient will not be allowed to be retreated with study therapy. After discontinuation of the investigational product, all patients will be followed for further anti-cancer treatments and survival until death. Further treatment of the patients may continue according to institutional standard of care.

Every effort will be made by the investigator to ensure that patients who have disease progression will continue to be followed up for the evaluation of the primary endpoint, even though they are no longer receiving investigational product.

## Primary efficacy dataset: Full Analysis Set (FAS)

Full analysis set (FAS) (primary analysis set) – all patients who are randomised to investigational product, excluding:

- all patients who do not receive at least one dose of investigational product;
- all patients who violate a major entry criterion; the major entry criteria are defined as follow:
  - Correct diagnosis defined by inclusion criteria 2: “Documented stage IV NSCLC as defined by IASLC Lung Cancer Staging Project (7th edition) or recurrent stage I-III disease at least 6 months after resection or after the end of adjuvant chemotherapy or after standard locoregional treatment as defined by the American College of Chest Physicians”
  - Disease control after the completion of platinum-based first-line chemotherapy defined by inclusion criteria 3: “Patients treated with 4 cycles platinum based 1st line chemotherapy as defined by the American College of Chest Physicians (i.e. radiotherapy are not allowed except palliative radiotherapy of bone metastasis)” and inclusion criteria 6: “CR, PR, or SD according to RECIST criteria after the completion of platinum-based first-line chemotherapy”
  - Correct laboratory conditions for eligibility of the trial therapy defined by inclusion criteria 4: “Documented HLA-A*0201 positivity, as determined by a local laboratory” and inclusion criteria 5: “TERT-positive NSCLC, as assessed by a central laboratory; for this, availability of adequate tissue biopsy from the primary tumor, lymph nodes or distant metastases is a prerequisite”

Patients in one or more of these 3 categories will only be excluded from the FAS if it can be clearly justified that their exclusion does not introduce bias. Patients in the FAS will be analysed according to the treatment administered at the first injection and their correct strata, provided that, in the case where the actual treatment/strata differ from the randomised treatment/strata, it can be clearly justified that this difference was not influenced by knowledge of the assigned treatment.

## Per-protocol (PP) dataset

Per-protocol (PP) analysis – all patients in the FAS who satisfy the following criteria:

- no major protocol deviations;
- good treatment compliance (early treatment discontinuations will only be considered for exclusion from the PP dataset if the reasons for early treatment discontinuation are unrelated to the assigned treatment).

## Safety (SAF) dataset

The safety dataset will include all patients who received at least one injection of investigational product.

In the event of subjects having received treatments that differed from those assigned according to the randomisation schedule, then the safety analyses should be conducted according to the treatment actually received (As Treated analysis) rather than according to the randomisation groups.

# Endpoints for analysis

## Efficacy endpoints

### Primary efficacy endpoint(s)

The primary endpoint of the study is the overall survival. Overall survival is defined as the time from the date of randomisation to the date of death.

### Secondary efficacy endpoints

Secondary endpoints of the study are:

- Survival rate at 12 months
- Time to Treatment failure

## Safety endpoints

The safety and tolerability will be evaluated by the monitoring of physical examinations, vital signs, electrocardiogram, the occurrence of AEs and SAEs, ECOG performance status, clinical laboratory evaluation and concomitant medication.

### Adverse events

Any untoward medical occurrence in a patient or clinical investigation patient administered a pharmaceutical product and which does not necessarily have to have a causal relationship with this treatment.

An Adverse event (AE) can therefore be any unfavorable and unintended sign (including an abnormal laboratory finding), symptom, or disease (new or exacerbated) temporally associated with the use of a medicinal product, whether or not considered related to the medicinal product. The events occurring during pre-and post-treatment periods will also be designated as AEs. Therefore, reporting of such events, AEs and SAEs, will commence when the patient is enrolled into the study (date of signature of the informed consent) up until 4 weeks after the end of the treatment visits.

Adverse events (AE) will be coded using the latest available version of the Medical Dictionary for Regulatory Activities (MedDRA) and will be classified by MedDRA Preferred Term (PT) and System Organ Class (SOC).

Treatment Emergent Adverse Events (TEAE) will be defined as any adverse event that occur or worsen after the start of the study treatment *i.e.:*

- An AE that was not present prior to receiving the beginning of study treatment, or
- An AE that was present prior to receiving beginning of study treatment but which made the condition worse after the study treatment started.

Serious adverse events (SAE) are those AE that put the life of the patient at risk or that cause serious or permanent damage to the patient's health. An AE is to be recorded and reported as SAE in the event of any of the following:

- AE results in death
- AE is life-threatening
- AE requires hospitalisation or prolongation of existing hospitalisation
- AE results in persistent or significant disability or incapacity
- AE is a congenital anomaly or birth defect.
- AE is another medically important condition: This refers to an AE that may not be immediately life-threatening or results in death or hospitalization but may jeopardize the patient or may require intervention to prevent one of the outcomes listed above. Based on medical and scientific judgment (according to the investigator and or the sponsor) this should usually be considered serious.

### Laboratory endpoints

The following clinical laboratory parameters will be measured at Baseline, week 3, week 6, week 9, week 12, week 15, week 18, every 12 week from week 27 to week 75 and every 24 week until End of treatment:

- Hematology: Hemoglobin, Hematocrit, WBC, Neutrophils, Lymphocytes, Monocytes, Eosinophils, Basophils and Platelet count
- Clinical chemistry: Sodium, Potassium, Calcium, Glucose, BUN or urea, Creatinine, AST, ALT, Alkaline phosphatase, Total bilirubin, Total protein, Albumin, LDH and Gamma-GT

### Other safety endpoints

#### **Physical examination and Vital signs**

A physical examination and vital signs will be conducted and recorded in the eCRF at Baseline, week 3, week 6, week 9, week 12, week 15, week 18, every 12 week from week 27 to week 75 and every 24 week until End of treatment.

Any physical examination abnormalities noted at baseline will be recorded and re-assessed at subsequent evaluations. All newly- diagnosed or worsening conditions or signs and symptoms observed after baseline evaluation, irrespectively of whether related to treatment or not, will be recorded as AEs.

Vital signs will include:

- blood pressure
- pulse rate after 5 minutes sitting,
- body temperature,
- weight, height (only at baseline).

#### **ECG**

A 12-lead ECG will be performed at baseline, week 18 and final visit (End of treatment visit)

#### **ECOG**

The ECOG performance status will be determined and recorded in the eCRF at Baseline, week 9, week 18, every 12 week from week 27 to week 75 and final visit.

The ECOG performance status will be graded according the following criteria:

- Grade 0: Fully active, able to carry on all pre-disease performance without restriction
- Grade 1: Restricted in physically strenuous activity, but ambulatory and able to carry out work of a light or sedentary nature, e.g., light house work, office work
- Grade 2: Ambulatory and capable of all selfcare, but unable to carry out any work activities. Up and about more than 50% of waking hours
- Grade 3: Capable of only limited self-care, confined to bed or chair more than 50% of waking hours
- Grade 4: Completely disabled. Cannot carry on any self-care. Totally confined to bed or chair
- Grade 5: Dead

#### **Concomitant medication**

Safety will be assessed based on the analysis of Concomitant medication coded according to the latest available version of the WHO-Drug dictionary.

# Statistical and Analytical Methods

## General considerations

The statistical analyses will be performed in accordance with the ICH E9 guideline and will be based on the pooled data from the individual study sites, unless otherwise stated.

The statistical analyses will be performed by an external Contract Research Organization (CRO), Venn Life Sciences, under the responsibility of the Sponsor. The full and detailed list of tables, listings and figures that will be produced is given in section 10.

### Presentation of results

The following statistics will be presented:

- For quantitative variables: number of available data, number of missing values, mean, standard deviation, median, Q1, Q3, minimum and maximum values. When relevant, confidence intervals will be calculated for the mean (Student CI) or the median (Hahn &  [Meeker](https://scholar.google.fr/citations?user=vB55GXIAAAAJ&hl=fr&oi=sra) 1991).
- For qualitative variables: number of available data, number of missing values, number and percentage of observations in each category of the variable. Except if otherwise specified, percentages will be calculated using the number of available data as denominator (i.e., not including missing values). When relevant, confidence intervals of proportions will be calculated using the Clopper-Pearson method (Clopper & Pearson 1934).

### Significance testing and estimation

The analyses of the primary efficacy criterion (overall survival) including the sensitivity analysis and of twelve months survival (secondary endpoint) will use a one-sided 0.05 type-one error rate. All other inferential tests on secondary or exploratory efficacy endpoints (see Sections 5.2.4.3 and 5.2.4.5) are presented only with an illustrative purpose and will use a two-sided 0.05 type-one error rate.

For some variables identified in this SAP, confidence intervals will be calculated with a two-sided coverage of 95%.

## Planned analysis

### Demographics and baseline characteristics

The following demographic variables will be summarised by treatment group on the FAS, SAF and the PP analysis data sets:

- Demographic data: Age and Gender and urine pregnancy test only for all women (Statistical Table 1, Listing 16.2.4.1)
- Disease characteristics (Results at screening visit for: Blood sample for HLA typing / Tumor sample for TERT and Chest/Abdomen MRI or CT / Bone scanning): HLA sample result, Biopsy sample result, scan type, scan result according to RECIST criteria (see Appendix 6: section 10.6), Bone scan, Bone scan result (Statistical Table 7, Listing 16.2.4.2):
- Medical history other than cancer under study by SOC and PT (Statistical table 10, Listing 16.2.4.5)
- Cancer history (Statistical Table 8, Listing 16.2.4.3)
- Treatment history (Statistical Table 9, Listing 16.2.4.4)

The following baseline characteristics will be described by treatment group on the FAS analysis data set:

- Laboratory test: Hematology at baseline (Statistical Table 11) and clinical chemistry at baseline (Statistical Table 12)
- Vital signs at baseline (Statistical Table 13)
- Physical examination at baseline (Statistical Table 14)
- 12–lead ECG at baseline (Statistical Table 15)
- ECOG performance status (Statistical Table 16)

### Patient disposition and study discontinuations

Patient disposition will be described using a listing (Listing 16.2.1.1), a statistical table (Statistical Table 2) and a flow chart (Figure 1).

The following variables will be tabulated for the screened population:

- Number of patients screened
- Number of patients included in the study
- Number of randomised patients, total and per treatment group
- Number of randomised patients assessed at each visit, total and per treatment group
- Number of randomised patients withdrawn from the study per reason for withdrawal, total and per treatment group

The numbers of patient within each dataset (FAS, PP and SAF), globally and by treatment group, will be provided (Statistical Table 3) together with numbers of patients assessed at each visit, globally and by treatment group. Reasons for exclusions from the FAS and PP populations will also be provided in Listing 16.2.3.1, according to ICHE3.

All protocol deviations will be tabulated by category (major or minor deviations, see Section 3.1) by treatment group for the randomized population (Statistical Table 5 and 6 respectively). All major and minor protocol deviations will also be provided in Listing 16.2.2.

### Extent of exposure and compliance

Compliance with study treatment will be computed for each subject of the FAS and the PP populations as the proportion of vaccinations actually performed (see section 8). Treatment compliance will then be analysed globally and by treatment group, as a numerical variable as well as a categorical variable (see section 8, Statistical Table 18).

To analyse the extent of exposure the following variables will be summarised by treatment group for the SAF population (Statistical Table 31):

- number of vaccinations performed
- duration (number of days) between first IP administration and last IP administration.

The number of patients administered investigational product will be summarized according to the duration of treatment, separately for each treatment groups and in total. The compliance and the duration of treatment will also listed in Listing 16.2.5.1 and Listing 16.2.5.2, respectively

### Efficacy analyses

#### Primary efficacy analysis

Initially, the primary endpoint of the study was overall survival at month 12. It was changed to overall survival following amendment Protocol dated 07 July 2015. Overall survival is defined as the time from the date of randomization to the date of death. For patients who will be alive at the time of analysis, overall survival will be censored at the time of the last available information data.

If no information is available on patient survival then every effort should be made by the investigator to obtain information on the patient vital status and the date of his/her death if relevant. In the rare cases where the information is missing, the patient will be censored at the time of the last information available.

The primary analysis (on the FAS set) will compare Overall survival using Kaplan Meier survival curves and Logrank test (Statistical Table 19, Figure 2). The primary analysis will be one-sided at the 0.05 significance level. Patients in the FAS will be analysed according to the treatment administered at the first injection and their correct strata, provided that, in the case where the actual treatment/strata differ from the randomised treatment/strata, it can be clearly justified that this difference was not influenced by knowledge of the assigned treatment.

#### Sensitivity efficacy analysis

To assess the robustness of the main analysis, several sensitivity analyses will be performed:

- The same primary analysis will be performed on the Per-Protocol set (Statistical Table 20, Figure 3).
- In case of randomisation errors (patients not receiving the treatment attributed by randomisation) and/or stratification errors (patients not attributed to the correct strata at the time of randomisation), the primary analysis will be repeated, according to treatment attributed by randomisation and strata used for the randomisation). If no patient show randomisation or stratification errors, this sensitivity analysis will not be applicable.
- Cox-model regressions of overall survival adjusted for the stratification factors and prognostic factors on the FAS and PP sets, a two-sided 90% confidence interval of the hazard ratio of the treatment effect will be provided (Statistical Table 21, Figure 4)
  - Stratification factors:
    - Response to first line chemotherapy: objective response vs. stable disease,
    - Squamous vs. non-squamous,
    - Stage IV vs. recurrent stage I-III
  - Prognostic factors:
    - Level of tumor TERT expression: low vs. high,
    - Vaccine specific immune response status: naturally occurring response vs. absence of naturally occurring response

#### Secondary efficacy analyses

The following secondary endpoints will be analysed on the FAS and PP sets:

- Twelve months survival:

A patient overall survival (success) at 12 months is defined as a patient who is alive as confirmed by the visit at week 52. If the patient misses the visit or has been lost to follow-up, every effort will be made by the investigator to ascertain whether the patient was alive on the theoretical date of his/her week 52 visit.

A one-sided Pearson’s chi-squared test of proportions will be used to compare the survival rate of the Vx-001 treated group to the placebo treated group at 12 months (Statistical Table 22).

- Time to Treatment Failure (TTF)

Time to treatment failure is defined as the interval between the date of first study treatment and the date of early withdrawal, or the date of the initiation of other anti-tumor treatment, whichever will come first. Patients without early withdrawal or initiation of another anti-tumor treatment will be censored at their last tumor assessment of target or non-target lesions. If patient have neither any failure events nor any post-baseline tumor assessment censoring time will be set to zero.

All these secondary endpoints are "time to event" endpoints and will be analyzed via

- Kaplan-Meier curves by treatment arm and log-rank test for their comparison (Statistical Table 23, Figure 5)
- Cox regression model where the treatment effect will be included and also the stratification factors and prognostic factors specified in the section 5.2.4.2 will be also included. Via this Cox model a two-sided 95% confidence interval of the hazard ratio of the treatment effect will be provided for both population (FAS and PP sets) (Statistical Table 24, Figure 6).

#### Exploratory efficacy analyses

The following endpoints will be analysed in various exploratory analyses:

- Disease Control of Subsequent Second Line Treatments

Disease control rate defined as the ratio of the number of patients who had confirmed response (PR or CR) or stable disease (SD) according to the RECIST 1.1 criteria to the number of patients in analysis population (FAS, Per-protocol set, resp.). The disease control rates after the end of subsequent second line treatments will be compared between Vx-001 treated and placebo treated patients via two-sided Pearson’s chi-squared test (Statistical Table 25).

- Vaccine Specific Immune Response

Vaccine specific immune response (determined by the percentage of ARG-Vx001 specific T cells producing IFN-γ and/or perforin) will be evaluated by IFN-γ ELISpot® assay and perforin ELISpot® assay performed at baseline, after the second and sixth injection or end of treatment in case of the patient withdrawal from the study before or at Week 18 and from week 39, every 24 weeks.

The impact of the vaccine induced immune response on overall survival will be studied as follows:

Comparison of overall survival in patients with vaccine induced immune response detected after the second and sixth injection of Vx-001 (Statistical Table 26, Statistical Table 27, Figure 7, and Figure 8):

- - patients who received at least two and six injections of placebo, respectively,
  - patients randomised to Vx-001 who did not develop vaccine specific immune response after the second and sixth injections of Vx-001, respectively.

Comparison of overall survival in Vx-001-treated vs placebo-treated patients who had (Statistical Table 28, Statistical Table 29, Figure 9, Figure 10):

- a vaccine specific immune response before Vx-001 or placebo injection,
- high level of TERT expression or low level of TERT expression in their tumour biopsy.

These comparisons will be done using Kaplan Meier survival curves and two-sided Logrank test on FAS set.

#### Other Exploratory Analyses

More general variants of Cox proportional hazards models will be applied to evaluate the influence of different prognostic factors, stratification factors, baseline characteristics on the treatment effect to time-to-death. Two-sided 95% confidence intervals of the hazard ratios of the treatment effect will be provided for each analysis on FAS set (Statistical Table 30, Figure 11). Additionally to stratification factors (Response to first line chemotherapy: objective response vs. stable disease, Squamous vs. non-squamous, Stage IV vs. recurrent stage I-III) and prognostic factors (Level of tumor TERT expression: low vs. high, Vaccine specific immune response status: naturally occurring response vs. absence of naturally occurring response) the following baseline characteristics will be included:

- - Age (at informed consent signing) : <65 and ≥65 years,
  - gender,
  - ECOG Performance Status,
  - smoking history,
  - First line chemotherapy (see section 8 for details on the grouping used).

### Safety analyses

Safety analysis will be based on the incidence, intensity, and type of adverse events, on clinical laboratory evaluations, physical exams, vital signs, ECG, ECOG performance status and local site reactions. Safety variables will be tabulated and presented for all subjects included in the Safety population (SAF).

#### Adverse events

**Adverse event listings:**

AE listings will be presented and sorted by subject, start date (calendar date and day from first vaccination administration), primary system organ class, preferred term and verbatim text for all adverse events recorded during the study and will include time since last vaccination and duration.

The following listings will be produced:

Listing of Adverse Events: (Listing 16.2.7.1) All adverse events for each patient, including the same event on several occasions, giving both preferred term and the original term used by the investigator. The listing will be sorted by site and by treatment group and should include: Patient identifier / Age, sex, weight, height / The adverse event (preferred term, reported term) / Duration of the adverse event / Severity according to NCI CTCAE / Seriousness (serious, non-serious) / Action taken with study treatment (none, permanent discontinuation, temporary discontinuation), / medication introduced / Outcome (resolved, resolved with sequelae, ongoing, lost-to-follow-up, fatal) / Causality relation to investigational treatment drug (not related, possible probable, definite) / Date of onset of the adverse event / Timing of onset of the adverse event.

Listings of Serious Adverse events, Adverse events leading to treatment discontinuation, Deaths, (Listing 16.2.7.2, Listing 16.2.7.3 and Listing 16.2.7.4 respectively), containing the same information as above

**Adverse events tabulations:**

Tabulation of adverse events will present for each cell the following information: number of patients with at least one occurrence of the event, corresponding percentage and number of events (if relevant). The following tables will be produced for all subjects included in the SAF population as well as by treatment group:

Summary table of adverse events (Statistical Table 32):

- Any AE
- Any TEAE
- Any drug-related TEAE
- Any TEAE leading to study drug discontinuation
- Any SAE
- Any treatment emergent SAE

For each category of the summary table of AE, 95% confidence intervals will be calculated for the incidence rates in each treatment group, together with a confidence interval for the treatment group difference in incidence rates.

A descriptive analysis will be performed for TEAEs. Global frequencies of SOC and PT will be calculated for:

- All TEAE by SOC an PT (Statistical Table 33)
- All not related TEAEs (*i.e.,* causality relationship to study drug is Not related) by SOC and PT (Statistical Table 34)
- All related TEAEs (*i.e.,* causality relationship to study drug is Possible, Probable or Definite) by SOC and PT (Statistical Table 35)
- All TEAEs by SOC and PT and by severity grade (Statistical Table 36)

Multiple counts within a PT or SOC (repeated or different included terms or changes in descriptors) will be considered only once per subject for the calculation of frequencies.

Depending on the number of TESAEs, similar tables as described above for TEAEs may be produced for TESAEs (Statistical Table 37, Statistical Table 38, Statistical Table 39 and Statistical Table 40). If the number of TESAEs is small, no specific tabulation of TESAEs will be produced.

#### Laboratory safety variables

Hematology and clinical chemistry data will be summarized at each scheduled assessment and presented by NCI CTCAE toxicity grade (version 4) (see Appendix 7: section 10.7).

In case of relevant abnormalities on the laboratory variables detected from statistics tabulations, the data will be presented graphically using a plot of mean level versus time by treatment group and/or a Pre/post treatment scattergram for each relevant laboratory variable (Figure 9 and 10 respectively).

**Tables on Laboratory safety variables:**

Quantitative descriptive statistics will be tabulated for each laboratory variable at each time over the course of the study (e.g., at each visit) on raw values and change from baseline (Statistical Table 41 and 44).

**Qualitative descriptive statistics:**

- Qualitative descriptions statistics will be tabulated for each laboratory variable at each time over the course of the study overall and by treatment group (Statistical Table 42 and 45):
  - By clinical significance (normal, abnormal NCS, abnormal CS)
  - By CTCAE grade
- Shift Tables between CTCAE grade or clinical significance for each laboratory results comparing baseline to each visit (Statistical Table 43 and 46).

A listing of all safety-related laboratory tests (Listing 16.2.8.1 and 16.2.8.3) will be prepared, presenting patient id, identification of time point, Age, Sex, weight and IMP dose, identification of laboratory test, result, flagged as abnormal when abnormal and normal range when available and relevant, toxicity grading when available. This listing will be presented by study site and by study treatment.

A patient listing of abnormal laboratory values will also presented (Listing 16.2.8.2 and 16.2.8.4)

#### Vital signs

For all vital signs recorded, quantitative descriptive statistics will be computed by visit (and treatment group on both raw values and change from baseline to each visit (Statistical table 47). All individual measurements will be provided in Listing 16.2.9.1.

#### **Physical examinations**

For all the variables collected during physical examinations, the frequencies of normal, abnormal NCS and abnormal CS values will be reported by visit and treatment group (Statistical table 48). All individual measurements will be provided in Listing 16.2.9.2.

#### **Injection site inspection**

Descriptive statistics for the global assessment of injection site completed by the investigator will be computed for each visit (at each treatment administration) by treatment group (Statistical table 49).

#### **ECG**

For 12-lead ECG assessments variables collected, the frequencies of normal, abnormal NCS and abnormal CS values will be reported by visit and treatment group (Statistical table 50). All individual measurements will be provided in Listing 16.2.9.3.

#### **ECOG performance status**

For ECOG performance status, the frequencies of each grade will be reported by visit and treatment group (Statistical table 51). All individual assessments will be provided in Listing 16.2.9.4.

#### **Prior and Concomitant medication**

Concomitant medication will be coded using the WHO DD thesaurus. Coding will be agreed upon with the sponsor before data base lock.

"Concomitant" is defined as a medication which was taken during treatment, e.g. stop date later than randomization date or start date before 4 weeks after end-of-treatment visit.

The medications reported prior to the baseline visit are:

- medications taken prior to randomization and related to NSCLC treatment (including response to treatment)
- medications taken in the 2 weeks prior to randomization and not related to NSCLC treatment,

Note: vitamins and/or dietary supplements are not considered concomitant medications.

Prior and concomitant medication will be tabulated by treatment group with the number and percentage of patients with prior medication classified by therapeutic class (level 2) and preferred name (Statistical table 17, Statistical table 52 respectively).

A by patient listing of concomitant medication will also be provided in Listing 16.2.10.4.

## Statistical/Analytical issues

### Adjustments for Covariates

All Cox models for all time-to-event endpoints, including for sensitivity analysis of the primary endpoint will be adjusted for the following stratification factors and prognostic factors:

- stratification factors:
  - Response to first line chemotherapy: objective response vs. stable disease,
  - Squamous vs. non-squamous,
  - Stage IV vs. recurrent stage I-III,
- prognostic factors:
  - Level of tumor TERT expression: low vs. high,
  - Vaccine specific immune response status: naturally occurring response vs. absence of naturally occurring response.

### Handling of Dropouts or Missing Data

For the primary endpoint, if no information is available on patient survival, the patient will be censored at the time of the last information available. Also, for all time-to-event analyses the same censoring approach will be used. For the 12 months survival analysis (secondary efficacy endpoint), the missing efficacy data will be imputed using the worst case approach *(i.e.* “death” for the patients randomised in the Vx-001 arm and “alive” for the patients randomised in the placebo arm).

For all other variables except missing/incomplete dates (see below), missing data will be treated as missing and no imputation will be done.

**Missing or incomplete dates**:

For all listings, missing or incomplete dates will be left as they were recorded.

For calculation / sorting / assignation based on dates (e.g. treatment emergent AEs, concomitant medications…), the following rules will apply:

- The most conservative approach will be considered (i.e. if the onset date of an AE/concomitant medication is missing / incomplete, it will be assumed to have occurred during the study treatment phase (*i.e.* a TEAE for AEs) except when the partial onset date or other available data indicate differently (*e.g.* start date day missing, but month before the month of baseline date, or stop date before baseline date).
- Medical history or disease diagnosis with missing/incomplete date will be assumed to have occurred before any study treatment except when the partial onset date or other available data indicates differently.
- Assignations based on dates will be reviewed and confirmed or infirmed during the data review meeting

### Interim Analyses and Data Monitoring

No Data and Safety Monitoring Board has been set-up for this study.

The primary study analysis will be performed when all patients randomised will have reached 6 months follow-up. A long term follow-up analysis will be performed later when the follow-up of the study will be completed.

Two database locks will be performed. The first lock will occur for the primary study analysis. Before this first database lock, the data will be reviewed and the classification of protocol deviations will be done during the Blind data review meeting. After the first database lock, all study personnel will have access to unblinded data.

### Multicentre studies

This study is conducted in 76 sites in 8 European Union countries. With regards to the large number of sites compared to the planned number of patients, no adjustment for centre effect will be done.

Nevertheless, a descriptive analysis of efficacy by country will be done for the primary efficacy analysis and the survival analysis at 12 months. The countries where the number of randomised patients is less than 10 will be pooled in order to describe overall survival and survival at 12 months by country.

### Multiple Comparison/Multiplicity

The primary efficacy analysis will be the only analysis used to draw definitive conclusions on efficacy for the study. As a single primary efficacy analysis has been clearly pre-specified, there is no need to adjust for multiplicity of analyses.

All other inferential tests will only be provided with an illustrative purpose and will not be used to “rescue” the study in case the primary analysis is non-significant.

Consequently all other inferential tests, except the sensitivity analyses described in Section 5.2.4.2 above will be presented with a two sided significance level of 0.05 and with two-sided 95% Confidence Intervals. The sensitivity analyses will use a one sided significance level of 0.05 and two-sided 90% Confidence Intervals, for consistency with the primary efficacy analysis.

### Use of an "Efficacy Subset" of Patients

Two efficacy analyses populations were defined, the FAS and the PP. The definition of these populations is given in Section 3. The primary efficacy analysis will be conducted on the FAS. The PP population will be used to conduct a sensitivity analysis and assess the robustness of the primary efficacy analysis conclusions. Any substantial difference between the two analyses will be explored and discussed.

Patients who did not receive at least one dose of investigational product and patients who violate a major entry criterion (as defined in section 3.2) will be exclude from the primary efficacy analysis

(Full analysis set, FAS) provided it can be clearly justified that their exclusion does not introduce bias.

### Active-Control Studies Intended to Show Equivalence

Not applicable.

### Examination of Subgroups

No specific subgroup analysis will be done but descriptive statistics will be presented on each stratification factors subgroups for the primary endpoint *i.e*.:

- - Response to first line chemotherapy: objective response
  - Response to first line chemotherapy: stable disease,
  - Squamous
  - non-squamous,
  - Stage IV
  - recurrent stage I-III,

The same descriptive analyses will be performed for each prognostic factors subgroups i.e.:

- - Level of tumor TERT expression: low
  - Level of tumor TERT expression high,
  - Vaccine specific immune response status: naturally occurring response
  - Vaccine specific immune response status: absence of naturally occurring response.

## Data handling conventions

### Baseline definitions

The measurements taken on baseline visit are assumed to be baseline values for the respective parameters.

Baseline visit, which takes place on day 1 of week 0, should be performed within 4 weeks after administration of the last cycle of first-line platinum-based chemotherapy.

The following characteristics will be measured at baseline visit:

- Physical examination and vital signs (blood pressure and pulse rate after 5 minutes sitting, body temperature and body weight, height)
- ECOG Performance Status.
- Electrocardiogram (12-lead ECG).
- Hematology.
- Clinical chemistry.
- Collection of blood sample for immunomonitoring. Samples will be collected before drug administration.

### Retest, Outliers

#### Retests

The retests will be managed as follow:

- Any retest before baseline : the last available value before baseline will be used as the baseline
- Any other retest: data will be reviewed and decision will be made during the blind review on the basis of the following rules:
  - Retest on efficacy data: non-missing value the closest to the scheduled visit will be used
  - Retest on safety data: the worst recorded value will be used

#### Outliers

All outlier data will be reviewed during the data review meeting and decisions regarding their use in the statistical analyses will be made.

### Windows for time points

Patients have to visit the clinic/institution for investigational product administration and assessments according to the Flow Chart and Schedule of Assessments (see section 2.3.3) Permitted deviations from the predetermined dates of visit (due to weekends, public holidays etc) may be as follows:

- Screening period: can start during the first line chemotherapy and should be completed at the latest 3 weeks and 4 days after administration of the last cycle of first-line platinum-based chemotherapy,
- Baseline, randomization and first dose of vaccination: up to 4 weeks and 4 days after the last administration of the first-line chemotherapy
- Doses 2-6 of vaccination: ± 4 calendar days
- Week 18 : ± 4 calendar days
- Doses 7 to end of study vaccination: ± 14 calendar days
- End of study treatment visit: ± 4 calendar days
- Survival follow-up contact: ± 28 calendar days

Any deviations greater than the above, will be regarded as a protocol deviation. The sponsor will have to be notified, to decide whether the deviation constitutes a minor or major protocol deviation. If required, the Steering committee will be consulted. When the permitted deviations are applied, or when the study treatment has to be postponed, the next study visit should be carried out at a time that allows a minimum of 14 days between 2 vaccinations (from doses 1 to 6 of vaccination) and a minimum of 2 months between 2 vaccinations (from doses 7 to the end of study treatment). In addition, effort should be made to keep up with the schedule, based on the date of first investigational product administration (first dose).

### Unscheduled visits

Unscheduled visit measurements may be used to provide a measurement for a baseline or endpoint value if appropriate according to their definition. Other unscheduled visits data will not be analysed, but corresponding data will be presented in the individual data listings and any relevant safety data will be described in the CSR.

# Modifications from the statistical sections in the protocol

Due to the initial slow enrolment, study results will only be available more than 2 years later than initially planned. In addition, patients randomized early in the trial have had a longer follow-up than they would have had with the planned duration of the study.

Provided that the recruitment was slower than anticipated, the initially planned primary efficacy criterion (12 months overall survival) and its planned analysis (comparison of proportions in the two treatment arms using a one-sided Pearson’s chi-squared test) are not anymore the optimal statistical approach in terms of statistical efficiency (power). The information on deaths occurring after the 12 months of follow-up in patients randomised early in the study and the time pattern of the deaths observed during the first year of follow-up are not taken into account with this approach.

Consequently, the primary endpoint has been changed to overall survival analysed with time to events methods (Kaplan Meier and Logrank test). This change should result in an increased power and will allow analysing the study before achieving 1 year follow-up for the last randomized patient.

As this analysis was already planned as a secondary analysis in the protocol and is widely accepted as a valid approach in cancer trials analysis, promoting it as the primary statistical analysis in this protocol is not a major change in the study aims and objectives (To demonstrate the clinical efficacy of Vx-001 vs placebo, as maintenance treatment after first-line treatment in NSCLC patients with disease control).

The initially planned primary efficacy analysis (comparison of 12 months overall survival rates in the two treatment arms using a one-sided a Pearson’s chi-squared test) will be presented as a secondary analysis.

Also, for consistency with the primary efficacy analysis, the significance level of the sensitivity analysis has been changed and will use a one-sided significance level of 0.05 and two-sided 90% Confidence Intervals.

| **Initial protocol** | **Change** |
| --- | --- |
| **Primary Objective:**  The primary objective is to compare survival rate at 12 months in Vx-001 treated vs placebo treated patients  **Secondary Objectives:**  Time-to-event comparison of overall survival in Vx-001 treated vs placebo treated patients.  **Exploratory Objectives:**  Comparison of survival rate at 12 months in patients with vaccine induced immune response detected after the second and sixth injection of Vx-001 *vs*  •patients who received at least two and six injections of placebo, respectively,  •patients randomised to Vx-001 who did not develop vaccine specific immune response after the second and sixth injections of Vx-001, respectively.  Comparison of survival rate at 12 months in Vx-001-treated *vs* placebo-treated patients who had:  •a vaccine specific immune response before Vx-001 or placebo injection,  •high levels of TERT expression or low levels of TERT expression in their tumour biopsy. | **Primary Objective:**  The primary objective is a time-to-event comparison of overall survival in Vx-001 treated vs placebo treated patients  **Secondary Objectives:**  Comparison of survival rate at 12 months in Vx-001 treated vs placebo treated patients.  **Exploratory Objectives:**  Comparison of overall survival in patients with vaccine induced immune response detected after the second and sixth injection of Vx-001 *vs*  •patients who received at least two and six injections of placebo, respectively,  •patients randomised to Vx-001 who did not develop vaccine specific immune response after the second and sixth injections of Vx-001, respectively.  Comparison of overall survival in Vx-001-treated vs placebo-treated patients who had:  •a vaccine specific immune response before Vx-001 or placebo injection,  •high levels of TERT expression or low levels of TERT expression in their tumour biopsy. |

The objective of the other exploratory analyses (5.2.4.5) is to evaluate the influence of different prognostic factors, stratification factors, baseline characteristics (age, gender, ECOG and smoking history) on the treatment effect to time-to-death using Cox proportional hazards models. The first line treatment at baseline is added to the list of factors explored in the Cox model because some of these first line therapies may have different impacts on the inflammatory and/or immune responses to the tested vaccine and hence influence the response to the vaccine.

# Software documentation

All summaries and statistical analyses will be generated using SAS version 9.2 or higher.

# Derived data

| **Derived variable** | **Derivation algorithm** |
| --- | --- |
| Change from baseline to visit V (continuous) | Change from baseline of variable X=X_(Visit V)_-X_(Baseline)_   - Negative values indicate a decrease in X - Positive values indicate an increase in X |
| Percent change from baseline to visit V (continuous) | Percent change from baseline of variable X=100 *[X_(Visit V)_-X_(Baseline)_]/ X_(Baseline)_   - Negative values indicate a decrease in X - Positive values indicate an increase in X |
| First line treatment chimiotherapy | CDDP or Cisplatin/Docetaxel or Paclitaxel  CDDP/Gemcitabine  CDDP/Pemetrexed  CDDP/Vinorelbine (Navelbine)  CDDP/Etoposide  Carboplatine/Docetaxel or Paclitaxel  Carboplatine/Gemcitabine  Carboplatine /Pemetrexed  Carboplatine/ Vinorelbine (Navelbine) |

# References

**Oken MM, Creech RH, Tormey DC, et al**. (1982) Toxicity and Response Criteria of the Eastern Cooperative Oncology Group. *Am J Clin Oncol* 61, 7196-7203.

**Fidias P.M., S.R. Dakhil, A.P. Lyss, D.M. Loesch, D.M. Waterhouse, J.L. Brommund, R. Chen et al.** (2009) Phase III study of immediate compared with delayed docetaxel after front-line therapy with gemcitabine plus carboplatin in advanced non-small-cell lund cancer. *J. Clin. Oncol.*, **27**: 591-98

**Cappuzzo F., T. Ciuleanu, L. Stelmackh, S. Cirenas, A. Szczesna, E. Juhacz, E. Esteban, O. Molinier, W. Brugger, I. Melezinek, GF. Klingelschmitt, B. Klunghammer, G. Giaccone** (2010). Erlotinib as maintenance treatment in advanced non small cell lung cancer: a multicenter, randomized, placebo controlled phase 3 study*. Lancet Oncol*., **11**, 521-29

**Ciuleanu T., T. Brodowicz, C. Zielinski, J.H. Kim, M. Kirzakowski et al.** Maintenance pemetrexed plus best supportive care *vs* placebo plus best supportive care for non-small-cell lung cancer: a randomized, double-blind, phase 3 study. Lancet, 2009, 374: 1432-40

# Appendices

## Appendix 1: Statistical Tables List

| **Demographics and Baseline** | |
| --- | --- |
| 1 | Demographics (11.2) |
| 2 | Disposition of patients (10.1) |
| 3 | Datasets analyzed (11.1) |
| 4 | Reasons for study treatment discontinuations |
| 5 | Major protocol deviations (10.2) |
| 6 | Minor protocol deviations (10.2) |
| 7 | Disease characteristics |
| 8 | Cancer history |
| 9 | Treatment Cancer history |
| 10 | Medical history |
| 11 | Hematology at baseline |
| 12 | Clinical chemistry at baseline |
| 13 | Vital Signs at baseline |
| 14 | Physical examination at baseline |
| 15 | ECG |
| 16 | ECOG performance status |
| 17 | Prior medication |
| 18 | Treatment compliance |
| **Efficacy Results** | |
| 19 | Primary efficacy analysis: Overall Survival |
| 20 | Sensitivity efficacy analysis: Overall Survival |
| 21 | Sensitivity efficacy analysis: Overall survival adjusted for the stratification factors and prognostic factors |
| 22 | Secondary efficacy analysis: Twelve months survival |
| 23 | Secondary efficacy analysis: Time to treatment failure |
| 24 | Secondary efficacy analysis: Time to treatment failure adjusted for the stratification factors and prognostic factors |
| 25 | Exploratory efficacy analysis: Disease Control of Subsequent Second Line Treatments - Comparison of disease control rate between treatment groups by Pearson's chi-squared test |
| 26 | Exploratory efficacy analysis: Vaccine Specific Immune Response - Comparison of overall survival with vaccine induced immune response detected after 2nd injection of Vx-001 vs. Patients randomised to Vx-001 who did not develop vaccine specific immune response after 2nd injections of Vx-001. |
| 27 | Exploratory efficacy analysis: Vaccine Specific Immune Response - Comparison of overall survival with vaccine induced immune response detected after 6th injection of Vx-001 vs. Patients randomised to Vx-001 who did not develop vaccine specific immune response after 6th injections of Vx-001. |
| 28 | Exploratory efficacy analysis: Vaccine Specific Immune Response - Comparison of overall survival in Vx-001-treated vs. Placebo treated patients who had a vaccine specific immune response before Vx-001 or placebo injection |
| 29 | Exploratory efficacy analysis: Vaccine Specific Immune Response- Comparison of overall survival in Vx-001-treated vs. Placebo-treated patients who had high levels of TERT expression or low levels of TERT expression in their primary tumors |
| 30 | Other exploratory efficacy analyses: More general variants of Cox proportional hazards models will be applied to evaluate the influence of different prognostic factors, stratification factors, baseline characteristics on the treatment effect to time-to-death |
| **Safety Results** | |
| 31 | Extent of exposure (12.1) |
| 32 | Summary of Adverse Events |
| 33 | All TEAEs by SOC and PT |
| 34 | All not related TEAEs by SOC and PT |
| 35 | All related TEAEs by SOC and PT |
| 36 | All TEAEs by SOC, PT and severity grade |
| 37 | All TESAEs by SOC and PT |
| 38 | All not related TESAEs by SOC and PT |
| 39 | All related TESAEs by SOC and PT |
| 40 | All TESAEs by SOC, PT and severity grade |
| 41 | Laboratory Tests - Hematology - Quantitative |
| 42 | Laboratory Tests - Hematology - Qualitative |
| 43 | Laboratory Tests - Hematology - Shift tables |
| 44 | Laboratory Tests - Chemistry - Quantitative |
| 45 | Laboratory Tests - Chemistry - Qualitative |
| 46 | Laboratory Tests - Chemistry - Shift tables |
| 47 | Vital signs |
| 48 | Physical examination |
| 49 | Injection site inspection |
| 50 | 12-lead ECG |
| 51 | ECOG performance status |
| 52 | Concomitant medication |

## Appendix 2: Statistical Figures List

| **Demographics and Baseline** | |
| --- | --- |
| 1 | Disposition of patients (10.1) |
| **Efficacy Results** | |
| 2 | **Primary efficacy analysis:** Overall Survival (Kaplan Meier curve) |
| 3 | **Sensitivity efficacy analysis:** Overall Survival (Kaplan Meier curve) |
| 4 | **Sensitivity efficacy analysis:** Overall survival adjusted for the stratification factors and prognostic factors |
| 5 | **Secondary efficacy analysis**: Time to treatment failure (Kaplan Meier curve) |
| 6 | **Sensitivity efficacy analysis:** Time to treatment failure adjusted for the stratification factors and prognostic factors |
| 7 | **Exploratory efficacy analysis:** Vaccine Specific Immune Response (Kaplan Meier curve) - Comparison of overall survival with vaccine induced immune response detected after 2nd injection of Vx-001 vs. Patients randomised to Vx-001 who did not develop vaccine specific immune response after 2nd injections of Vx-001. |
| 8 | **Exploratory efficacy analysis**: Vaccine Specific Immune Response (Kaplan Meier curve) - Comparison of overall survival with vaccine induced immune response detected after 6th injection of Vx-001 vs. Patients randomised to Vx-001 who did not develop vaccine specific immune response after 6th injections of Vx-001. |
| 9 | **Exploratory efficacy analysis:** Vaccine Specific Immune Response (Kaplan Meier curve) - Comparison of overall survival in Vx-001-treated vs. Placebo treated patients who had a vaccine specific immune response before Vx-001 or placebo injection |
| 10 | **Exploratory efficacy analysis:** Vaccine Specific Immune Response (Kaplan Meier curve) - Comparison of overall survival in Vx-001-treated vs. Placebo-treated patients who had high levels of TERT expression or low levels of TERT expression in their primary tumors |
| 11 | **Other exploratory efficacy analysis:** Overall survival adjusted for the stratification factors, prognostic factors and baseline characteristics |
| **Safety results** | |
| 12 | Pre/Post Treatment scattergram for laboratory variable |
| 13 | Plot of mean level versus time by treatment group |

## Appendix 3: Individual Data Listings List

| **16.2.1** | **Discontinued Patients** |
| --- | --- |
| 16.2.1.1 | Disposition of patients - All subjects |
| 16.2.1.2 | Disposition of patients (10.1) / Discontinued patients |
| **16.2.2** | **Protocol deviations** |
| 16.2.2 | Protocol deviations (10.2) |
| **16.2.3** | **Patients excluded from the efficacy analysis** |
| 16.2.3.1 | Patients excluded from the efficacy analysis |
| 16.2.3.2 | Datasets analysed |
| 16.2.4 | **Demographic data** |
| 16.2.4.1 | Patients characteristics |
| 16.2.4.2 | Disease characteristics |
| 16.2.4.3 | Cancer history |
| 16.2.4.4 | Treatment Cancer history |
| 16.2.4.5 | Medical History |
| 16.2.4.6 | Prior medication |
| **16.2.5** | **Compliance data** |
| 16.2.5.1 | Treatment administration and exposure |
| 16.2.5.2 | Compliance |
| **16.2.6** | **Individual efficacy response data** |
| 16.2.6.1 | Primary endpoint data - Overall survival |
| 16.2.6.2 | Secondary endpoint - Time to treatment failure |
| 16.2.6.3 | Secondary endpoint - Twelve months survival |
| 16.2.6.4 | Exploratory endpoint - Disease Control of Subsequent Second Line Treatments |
| **16.2.7** | **Adverse events listings** |
| 16.2.7.1 | Adverse events |
| 16.2.7.2 | Serious Adverse events |
| 16.2.7.3 | Adverse Events leading to treatment discontinuation |
| 16.2.7.4 | Deaths |
| **16.2.8** | **Individual laboratory measurments** |
| 16.2.8.1 | Laboratory tests - Hematology |
| 16.2.8.2 | Abnormal Laboratory tests - Hematology |
| 16.2.8.3 | Laboratory tests - Chemistry |
| 16.2.8.4 | Abnormal Laboratory tests - Chemistry |
| **16.2.9** | **Other safety data** |
| 16.2.9.1 | Vital signs |
| 16.2.9.2 | Physical examination |
| 16.2.9.3 | 12-lead ECG |
| 16.2.9.4 | ECOG performance status |
| **16.2.10** | **Other listings** |
| 16.2.10.1 | Informed consent |
| 16.2.10.2 | Inclusion/Exclusion criteria |
| 16.2.10.3 | Date of visit |
| 16.2.10.4 | Concomitant medication |

## Appendix 4: TNM Clinical Classification of Lung Cancer

The TNM staging characterizes lung cancer with respect to several factors which include tumor size and location, extent of lymphatic invasion and the presence of distant metastases.

The current version of the staging system used has been published in *AJCC Cancer Staging Manual, 7^th^ edition*:

**Primary Tumor (T)**

**TX:** Primary tumor cannot be assessed, or tumor proven by the presence of malignant cells in sputum or bronchial washings but not visualized by imaging or bronchoscopy

**TX:** Primary tumor cannot be assessed, or tumor proven by the presence of malignant cells in sputum or bronchial washings but not visualized by imaging or bronchoscopy

**T0:** No evidence of primary tumor

**Tis:** Carcinoma in situ

**T1:** Tumor 3 cm or less in greatest dimension, surrounded by lung or visceral pleura, without bronchoscopic evidence of invasion more proximal than the lobar bronchus (for example, not in the main bronchus)^1^

**T1a:** Tumor 2 cm or less in greatest dimension

**T1b:** Tumor more than 2 cm but 3 cm or less in greatest dimension

**T2:** Tumor more than 3 cm but 7 cm or less or tumor with any of the following features (T2 tumors with these features are classified T2a if 5 cm or less): involves main bronchus, 2 cm or more distal to the carina; invades visceral pleura (PL1 or PL2); associated with atelectasis or obstructive pneumonitis that extends to the hilar region but does not involve the entire lung

**T2a:** Tumor more than 3 cm but 5 cm or less in greatest dimension

**T2b:** Tumor more than 5 cm but 7 cm or less in greatest dimension

**T3:** Tumor more than 7 cm or one that directly invades any of the following: parietal pleural (PL3), chest wall (including superior sulcus tumors), diaphragm, phrenic nerve, mediastinal pleura, parietal pericardium; or tumor in the main bronchus less than 2 cm distal to the carina1 but without involvement of the carina; or associated atelectasis or obstructive pneumonitis of the entire lung or separate tumor nodule(s) in the same lobe

**T4:** Tumor of any size that invades any of the following: mediastinum, heart, great vessels, trachea, recurrent laryngeal nerve, esophagus, vertebral body, carina, separate tumor nodule(s) in a different ipsilateral lobeDistant Metastasis (M) M0 No distant metastasis M1 Distant metastasis M1a Separate tumor nodule(s) in a contralateral lobe, tumor with pleural nodules or malignant pleural (or pericardial) effusion2 M1b Distant metastasis (in extrathoracic organs)

**Distant Metastasis (M)**

**M0:** No distant metastasis

**M1:** Distant metastasis present

**M1a: Separate tumor nodule(s) in a contralateral lobe, tumor with pleural nodules or malignant pleural (or pericardial) effision^2^**

**M1b: Distant metastasis (in extrathoracic organs)**

^1^ Note: The uncommon superficial spreading tumor of any size with its invasive component limited to the bronchial wall, which may extend proximally to the main bronchus, is also classified as T1a.

^2^ Note: Most pleural (and pericardial) effusions with lung cancer are due to tumor. In a few patients, however, multiple cytopathologic examinations of pleural (pericardial) fluid are negative for tumor, and the fluid is nonbloody and is not an exudate. Where these elements and clinical judgment dictate that the effusion is not related to the tumor, the effusion should be excluded as a staging element and the patient should be classified as M0.

**Regional Lymph Nodes (N)**

The regional lymph nodes are the intrathoracic, scalene and supraclavicular nodes

**NX:** Regional lymph nodes cannot be assessed

**N0:** No regional lymph node metastasis

**N1:** Metastasis in ipsilateral peribronchial and/or ipsilateral hilar lymph nodes and intrapulmonary nodes, including involvement by direct extension

**N2:** Metastasis in ipsilateral mediastinal and/or subcranial lymph node(s)

**N3:** Metastasis in contralateral mediastinal, contralateral hilar, ipsilateral or contralateral scalene, or supraclavicular lymph node(s)

**AJCC Stage Groupings**

| **ANATOMIC STAGE / PROGNOSTIC GROUPS** | | | |
| --- | --- | --- | --- |
| Occult carcinoma | TX | N0 | M0 |
| Stage 0 | Tis | N0 | M0 |
| Stage 1A | T1a | N0 | M0 |
|  | T1b | N0 | M0 |
| Stage 1B | T2a | N0 | M0 |
| Stage IIA | T2b | N0 | M0 |
|  | T1a | N1 | M0 |
|  | T1b | N1 | M0 |
|  | T2a | N1 | M0 |
| Stage IIB | T2b | N1 | M0 |
|  | T3 | N0 | M0 |
| Stage IIIA | T1a | N2 | M0 |
|  | T1b | N2 | M0 |
|  | T2a | N2 | M0 |
|  | T2b | N2 | M0 |
|  | T3 | N1 | M0 |
|  | T3 | N2 | M0 |
|  | T4 | N0 | M0 |
|  | T4 | N1 | M0 |
| Stage IIIB | T1a | N3 | M0 |
|  | T1b | N3 | M0 |
|  | T2a | N3 | M0 |
|  | T2b | N3 | M0 |
|  | T3 | N3 | M0 |
|  | T4 | N2 | M0 |
|  | T4 | N3 | M0 |
| Stage IV | Any T | Any N | M1a |
|  | Any T | Any N | M1b |

## Appendix 5: ECOG Performance Status

| **Grade** | **Eastern Cooperative Oncology Group (ECOG)** |
| --- | --- |
| 0 | Fully active, able to carry on all pre-disease performance without restriction |
| 1 | Restricted in physically strenuous activity, but ambulatory and able to carry out work of a light or sedentary nature, *e.g.*, light house work, office work |
| 2 | Ambulatory and capable of all selfcare, but unable to carry out any work activities. Up and about more than 50% of waking hours |
| 3 | Capable of only limited self-care, confined to bed or chair more than 50% of waking hours |
| 4 | Completely disabled. Cannot carry on any self-care. Totally confined to bed or chair |
| 5 | Dead |

Oken et al. 1982

## Appendix 6: Revised RECIST guideline (version 1.1)

(Eisenhauer E.A. *et al*., 2009)

**I. Definition of Measurable and Non-Measurable Disease**

- **Measurable disease:** Presence of at least one measurable lesion
- **Measurable lesion (non-nodal):** A lesion that can be accurately measured in at least one dimension (longest diameter; LD) with a minimum size of:
- ≥10 mm by CT scan (assuming CT slice thickness is no greater than 5 mm). If CT slice thickness is greater than 5 mm, the minimum size of the lesion should be twice the slice thickness.
- 10 mm measurable with calipers by clinical exam (if lesions cannot be accurately measured with calipers they should be recorded as non-measurable)
- 20 mm by chest X-ray
- **Lymph nodes:** measured in short axis
- Target lesions: to be considered pathologically enlarged and measurable, lymph nodes should be ≥15 mm in short axis as assessed by CT scan (CT slice thickness no greater than 5mm)
- Non-target lesions: pathological nodes with short axis ≥10 mm but <15 mm
- Non-pathological: nodes with short axis <10 mm
- **Non-measurable lesion:**
- Small lesions with longest diameter <10 mm
- Pathological lymph nodes with ≥10 to <15 mm short axis
- Blastic bone lesions
- Truly non-measurable lesions: include leptomeningeal disease, ascite, pleural or pericardial effusion, inflammatory breast disease, lymphangitic involvement of skin or lung, abdominal masses/abdominal organomegaly identified by physical exam that is not measurable by reproducible imaging techniques.

**II. Methods of Measurement**

The same method of assessment and the same technique should be used to characterize each identified and reported lesion at baseline and during follow-up.

**Computer Tomography (CT) or Magnetic Resonance Imaging (MRI):** CT is considered the best currently available and reproducible method. Measurability of lesions on CT scans is based on the assumption that CT scans are performed at 5 mm contiguous slice thickness or less. If slice thickness is greater than 5 mm, the measurable lesion should have a minimum size of twice the slice thickness.

MRI can be acceptable in certain situations. When MRI is performed, the technical specification of the scanning sequences used should be optimized for the evaluation of the type and site of disease. The lesions must be measured on the same pulse sequence and the modality used at follow up should be the same as baseline. Ideally, the same scanner should be used and the image acquisition protocol should be followed as closely as possible to prior scans.

Please note the following:

- All measurements should be taken and recorded in metric notation, using a ruler or calipers.
- Ultrasound is not an acceptable method of measurement.
- Tumor markers alone cannot be used to assess objective tumor response.

**III. Baseline Documentation of Target and Non-Target Lesions**

- Target lesions should be selected on the basis of their size (lesions with LD), they should be representative of all involved organs and lend themselves to reproducible repeated measurements (if largest lesion does not lend itself to reproducible measurement, the next largest which can be measured reproducibly should be selected). All measurable lesions up to a **maximum of 2 lesions per organ and 5 lesions in total, representative of all involved organs**, should be identified as target lesions and measured and recorded at baseline.

**Pathological lymph nodes, defined as measurable and identified as** target lesions should be ≥15 mm in short axis by CT scan. Pathological nodes are considered non-target lesions with short axis ≥10 mm but <15 mm. Non-pathological are nodes with short axis <10 mm and should not be recorded or followed.

- A sum of the diameters, longest for non-nodal, short axis for nodal lesions, for all target lesions will be calculated and reported as the baseline sum diameters. For lymph nodes, only the short axis is added into the sum. For PR, SD and PD the actual short axis measurement of the nodes is to be included in the sum of target lesions. The baseline sum diameters will be used as a reference by which to characterize the objective tumor response.
- All other lesions (or sites of disease) including pathological lymph nodes should be identified as non-target lesions and should also be recorded at baseline. Measurements of these lesions are not required, but they should be followed as “present” or “absent” or in rare cases “unequivocal progression”. In addition, it is possible to record multiple non-target lesions involving the same organ as a single item on the case record form.

**IV. Response Criteria**

**Evaluation of Target Lesions**

The definitions for assessment of response for target lesion(s) are as follows:

- **Complete Response (CR)** – disappearance of all target lesions. Any pathological lymph nodes (whether target or non-target) must have reduction in short axis to <10 mm.
- **Partial Response (PR)** – at least a 30% decrease in the sum of the diameters of target lesions, taking as a reference the baseline sum diameters.
- **Stable Disease (SD)** – neither sufficient shrinkage to qualify for PR nor sufficient increase to qualify for progressive disease (PD), taking as reference, the smallest sum diameters since the treatment started.
- **Progressive Disease (PD)** – at least a 20% increase in the sum of diameters of target lesions, taking as a reference, the smallest sum on study (this included the baseline sum if that is the smallest on study). In addition to the relative increase of 20%, the sum must also demonstrate an absolute increase of at least 5 mm.

The appearance of one or more new lesions is also considered progression.

- **Not-evaluated (NE)** – any patient who cannot be classified by any of the above four definitions.

**Evaluation of Non-Target Lesions**

The definitions for assessment of objective tumor response for non-target lesions are as follows:

- **Complete Response (CR)** – the disappearance of all non-target lesions and normalization of tumor marker level. All lymph nodes must be non-pathological in size (<10 mm short axis).
- **Non-CR/Non-PD** – the persistence of one or more non-target lesion(s) and/or maintenance of tumor marker level above the normal limits.
- **Progressive Disease (PD)** – Unequivocal progression of existing non-target lesions. The appearance of one or more new lesions is also considered progression.

Note: “Unequivocal progression” on the basis of non-target disease when the patient also has measurable disease, indicates an overall level of substantial worsening in non-target disease such that even in presence of SD or PR in target disease, the overall tumor burden has increased sufficiently and therapy should be stopped. A modest increase in the size of one or more non-target lesions is usually non-sufficient.

**Evaluation of new lesions**

- The finding of a new lesion should be unequivocal, *i.e.* not attributable to different scanning technique or findings thought to represent something other tan tumor.
- When the lesion is equivocal, therapy should be continued and follow-up evaluation will clarify if it represents truly new disease (if yes progression has the date of the initial scan).
- If a new lesion is identified in an anatomical location that was not scanned at baseline, it is considered a new lesion and indicates disease progression.

**Evaluation of Overall Response**

The following tables provide a summary of the overall response status calculation at each time point for patients who have measurable disease at baseline and non-measurable disease only.

Table 1: Time point response: patients with target (+/- non-target) disease

| **Target Lesions** | **Non-Target Lesions** | **New Lesions** | **Overall Response** |
| --- | --- | --- | --- |
| CR | CR | No | CR |
| CR | Non-CR/non-PD | No | PR |
| CR | Not evaluated | No | PR |
| PR | Non-PD or not all evaluated | No | PR |
| SD | Non-PD or not all evaluated | No | SD |
| Not all evaluated | Non-PD | No | NE |
| PD | Any | Yes or No | PD |
| Any | PD | Yes or No | PD |
| Any | Any | Yes | PD |

Table 2: Time point response: patients with non target disease only

| **Non-Target Lesions** | **New Lesions** | **Overall Response** |
| --- | --- | --- |
| CR | No | CR |
| Non-CR/non-PD | No | Non-CR/non-PD |
| Not all evaluated | No | NE |
| Unequivocal PD | Yes or No | PD |
| Any | Yes | PD |

CR=complete response, PR=partial response, SD =stable disease, PD=progressive disease, NE=inevaluable

**Evaluation of Best Overall Response**

The best overall response is the best response recorded from the start of treatment until disease progression/recurrence (taking as reference for PD, the smallest measurements recorded since treatment was initiated). In general, the patient's best overall response assignment will depend on the achievement of both measurement and confirmation criteria. If no confirmation of complete or partial response is required, best response is defined as the best response across all time points.

Patients with a global deterioration of health status requiring discontinuation of treatment without objective evidence of disease progression at that time should be classified as having "symptomatic deterioration." Every effort should be made to document the objective progression by evaluation of target and non-target disease, even after discontinuation of treatment.

In some circumstances, it may be difficult to distinguish residual disease from normal tissue. When the evaluation of complete response depends on this determination, it is recommended that the residual lesion be investigated (fine needle aspirate/biopsy) to confirm the complete response status.

For equivocal findings on progression, treatment may continue until the next scheduled assessment. If progression is confirmed at the next assessment, the date of progression should be the earlier date when progression was suspected.

**Confirmation criteria**

Confirmation is only required in studies where objective response rate is the primary end-point. In all other circumstances, confirmation of response is not required.

## Appendix 7: U.S. National Cancer Institute (NCI) Common Terminology Criteria for Adverse Events (CTCAE)

The severity of adverse events will be assessed using the Common Terminology Criteria for Adverse Events (CTCAE), version 4.0. A copy can be downloaded from the internet web site:

http://ctep.cancer.gov/forms/CTC
